# Characterization of a novel AICARFT inhibitor which potently elevates ZMP and has anti-tumor activity in murine models

**DOI:** 10.1038/s41598-018-33453-4

**Published:** 2018-10-18

**Authors:** Harold B. Brooks, Timothy I. Meier, Sandaruwan Geeganage, Kevin R. Fales, Kenneth J. Thrasher, Susan A. Konicek, Charles D. Spencer, Stefan Thibodeaux, Robert T. Foreman, Yu-Hua Hui, Kenneth D. Roth, Yue-Wei Qian, Tao Wang, Shuang Luo, Alicia Torrado, Chong Si, James L. Toth, Jefferson R. Mc Cowan, Kwame Frimpong, Matthew R. Lee, Robert D. Dally, Timothy A. Shepherd, Timothy B. Durham, Yong Wang, Zhipei Wu, Philip W. Iversen, F. George Njoroge

**Affiliations:** Eli Lilly and Company, Lilly Research Laboratories, Indianapolis, Indiana, 46285 USA

## Abstract

AICARFT is a folate dependent catalytic site within the ATIC gene, part of the purine biosynthetic pathway, a pathway frequently upregulated in cancers. LSN3213128 is a potent (16 nM) anti-folate inhibitor of AICARFT and selective relative to TS, SHMT1, MTHFD1, MTHFD2 and MTHFD2L. Increases in ZMP, accompanied by activation of AMPK and cell growth inhibition, were observed with treatment of LY3213128. These effects on ZMP and proliferation were dependent on folate levels. In human breast MDA-MB-231met2 and lung NCI-H460 cell lines, growth inhibition was rescued by hypoxanthine, but not in the A9 murine cell line which is deficient in purine salvage. In athymic nude mice, LSN3213128 robustly elevates ZMP in MDA-MB-231met2, NCI-H460 and A9 tumors in a time and dose dependent manner. Significant tumor growth inhibition in human breast MDA-MB231met2 and lung NCI-H460 xenografts and in the syngeneic A9 tumor model were observed with oral administration of LSN3213128. Strikingly, AMPK appeared activated within the tumors and did not change even at high levels of intratumoral ZMP after weeks of dosing. These results support the evaluation of LSN3213128 as an antineoplastic agent.

## Introduction

**Figure 1:**
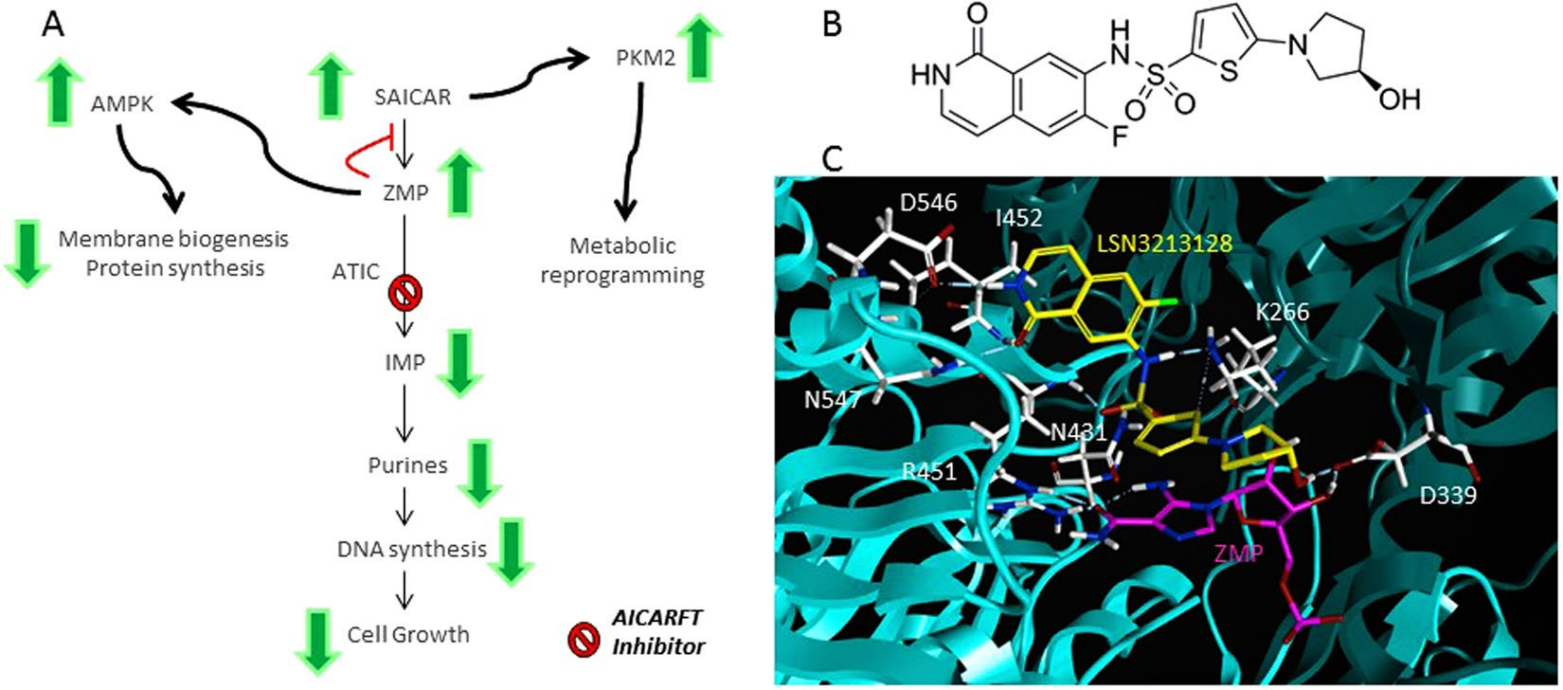
(**A**) Schematic of purine biosynthesis and the potential roles for ZMP as a signaling molecule, notably AMPK activation and SAICAR reported activation of PKM2. (**B**) The structure of LSN3213128. (**C**) Panel C illustrates a ribbon diagram of the homodimeric bifunctional protein encoded by the homodimeric ATIC with one monomer in cyan and the other in teal is shown in complex with 5-aminoimidazole-4-carboxyamide ribonucleotide (ZMP) in magenta and LSN3213128 in yellow. Only one formyl transferase active site of the homodimeric bifunctional protein is illustrated. Amino acids which hydrogen bond to LSN3213128 are shown in white. I452, D546 and N547 interact with the isoquinolone. K266, N431 & R451 interact with the sulfonamide. D339 interacts with the hydroxypyrrolidine. Both F541 and G316 make significant van der Waals contacts but are not shown for sake of clarity.

Pemetrexed is a classical anti-folate that inhibits thymidylate synthase (TS), dihydrofolate reductase (DHFR), glycinamide ribonucleotide formyltransferase (GARFT) and 5-aminoimidazole 4-carboxamide ribonucleotide transformylase inosine monophosphate cyclohydrolase (ATIC)^1^. GARFT and ATIC enzymes are required for purine biosynthesis. Purines are bases incorporated into both DNA and RNA, thus essential for cell proliferation^2^. Further investigation of pemetrexed showed that the inhibition of ATIC by pemetrexed leads to elevation of 5-aminoimidazole 4-carboxamide ribonucleotide (ZMP) and the activation of AMP-activated Protein Kinase (AMPK), suggesting that effects of pemetrexed on the ZMP/AMPK pathway may contribute to its anti-tumor activity(Fig. 1A)^3^. ZMP elevation using low dose methotrexate, which inhibits ATIC, has also been observed^4^.

The ZMP intermediate in purine biosynthesis and its metabolite, 5-aminoimidazole 4-carboxamide ribonucleoside (AICAR), is remarkable because ZMP is an energy sensor^5^. ZMP biosynthesis is the result of hydrolysis of succinyl-AICAR by adenylsuccinate lyase^6^. ZMP is converted to IMP by AICAR-transformylase inosine monophosphate cyclohydrolase (ATIC) which contains two catalytic sites, the AICAR-transformylase (AICARFT) site which uses 10-formyl tetrahydrofolate (THF) as a co-substrate and the inosine monophosphate cyclohydrolase (IMPCH) site^7^. In 2007 AICAR was suggested as a treatment for leukemia^8^. In 2008 AICAR was labeled as an “exercise mimetic” and considered a promising drug candidate for obesity and type-2 diabetes^9^. AICAR entered clinical trials for chronic lymphoid leukemia, demonstrating that AICAR administered by infusion was rapidly converted to ZMP^10^. Binding of ZMP to the AMPK γ subunit allows phosphorylation and activation of AMPK by LKB1^11^.

AMP-dependent protein kinase (AMPK) is a heterotrimeric protein that contains an α subunit which is a protein kinase, a scaffolding β subunit and a γ domain regulatory subunit^11^. Activated AMPK phosphorylates PCG-1α, HDAC, TSC1/2, Raptor and ACC1^11^. PCG-1α and HDAC are transcriptional coactivators activated by AMPK and regulate glucose metabolism. TSC1/2 and Raptor regulate protein synthesis via the TORC1 complex, thus AMPK inhibits eIF4E dependent protein translation. ACC1 is directly involved in lipid biosynthesis and is inhibited by AMPK phosphorylation. AMPK has emerged as central regulator of energy homeostasis^12^.

ATIC is an unusual homodimeric enzyme in that it contains two active sites^13^. The AICARFT site is formed at the interface between the homodimers and binds 10-formyl-THF and AICAR to produce FAICAR, an unstable intermediate. The IMPCH site catalyzes the cyclization of FAICAR to form IMP. Crystal structures of classical ATIC inhibitors such as BW2315 have been published^14^; however, their use in animal models is limited.

In order to test the hypothesis that the inhibition of purine biosynthesis with concomitant AMPK activation via ZMP will lead to anti-tumor efficacy, we developed a non-classical anti-folate, LSN3213128, as a novel and selective inhibitor of AICARFT^15^. Elevated ZMP and anti-proliferative effects in both tissue culture and *in vivo* models were observed with treatment of this orally bioavailable compound. LSN3213128 is used to explore the consequence of ZMP elevation in solid tumors.

## Results

LSN3213128 (Fig. 1B) is a potent folate inhibitor of AICARFT which binds in the folate binding pocket with ZMP (Fig. 1C) resulting in an IC50 of 16 ± 11 nM for the conversion of ZMP to IMP. This molecule has a sulfonamide group that binds to the oxyanion hole similar to BW2315^14^; however, LSN3213128 has a novel isoquinoline ring system replacing the pteridine moiety found in folic acid. Additionally, LSN3213128 uses a novel thiophene to replace the benzoate and a hydroxypyrrolidine to replace the glutamate. This compound is selective for AICARFT with IC50s >100 μM against TS, SHMT1, MTHFD1, MTHFD2 and MTHFD2L (Supplemental Table 1). The compound has been profiled in a panel of protein kinase assays at CEREP Panlabs (Eurofins) and has no significant protein kinase activity (Supplemental Table 2).

Due to pemetrexed’s antineoplastic effects in nonsquamous NSCLC and Moran’s identification of AICARFT as a secondary target in NCI-H460 cell line^16^, this lung cell line was selected to develop a cell based ZMP assay. Interestingly, NCI-H460 is mutant for LKB1. Table 1 shows both the ZMP response and cell growth inhibition measured using Alamar Blue for LSN3213128 in low folate RPMI media and in RPMI media. LSN3213128 appears to be a folate competitive inhibitor in tissue culture as seen by the lower ZMP EC50 in low folate media (average EC50 4.9 ± 2.6 nM for 12 cell lines) compared to normal RPMI (average EC50 1,210 ± 1,080 nM for 8 cell lines). For all cell lines tested, a similar trend was observed for the anti-proliferative response (Table 1) with average GI50 4.4 ± 4.5 nM (12 cell lines) in low folate shifting to an average GI50 of 2,400 ± 1,400 nM (11 cell lines) in regular RPMI. The shift between the biochemical assay and the cell assay in RPMI media is likely a consequence of 10-formylTHF polyglutamation. The enzymatic assay uses a monoglutamated substrate whereas the cellular 10-formylTHF is polyglutamated and lowers the Km for the substrate. Consequently the intracellular ZMP EC50 is higher than the enzymatic IC50 due to competition with polyglutamated 10-formylTHF.

**Table 1:** LSN3213128 activity in tissue culture.

Cell line	ZMP EC50 (nM) Low folate	ZMP EC50 (nM) Regular folate	Alamar Blue GI50 (nM) Low folate	Alamar Blue GI50 (nM) Regular folate
HCT 116 (colon)	2.7 ± 3.3	369 ± 44	2.2 ± 2.1	2811 ± 1220
SW620 (colon)	4.3 ± 0.9	600	8.8 ± 1.6	3910 ± 3239
SNU-16 (gastric)	2	2500	1.2 ± 1.2	1590 ± 1273
A549 (lung)	10	3100	6	—
NCI-H1155 (lung)	2.3	380	4.9	729 ± 215
NCI-H1299	4.1	—	4.8	1590
NCI-H1437 (lung)	4.6	620 ± 354	0.73	1883 ± 894
NCI-H1993 (lung)	7.1	—	3.0 ± 2.7	2676 ± 2537
NCI-H460 (lung)	8	428 ± 102	16.4 ± 5.1	3470 ± 2092
MDA-MB-231 (mammary)	7.6	89	0.49	85 ± 42
A101D (melenoma)	2.3	—	2.9	3290
MIA PaCa-2 (pancreas)	4.2	1700	1.6 ± 1.0	4870 ± 4200

**Figure 2:**
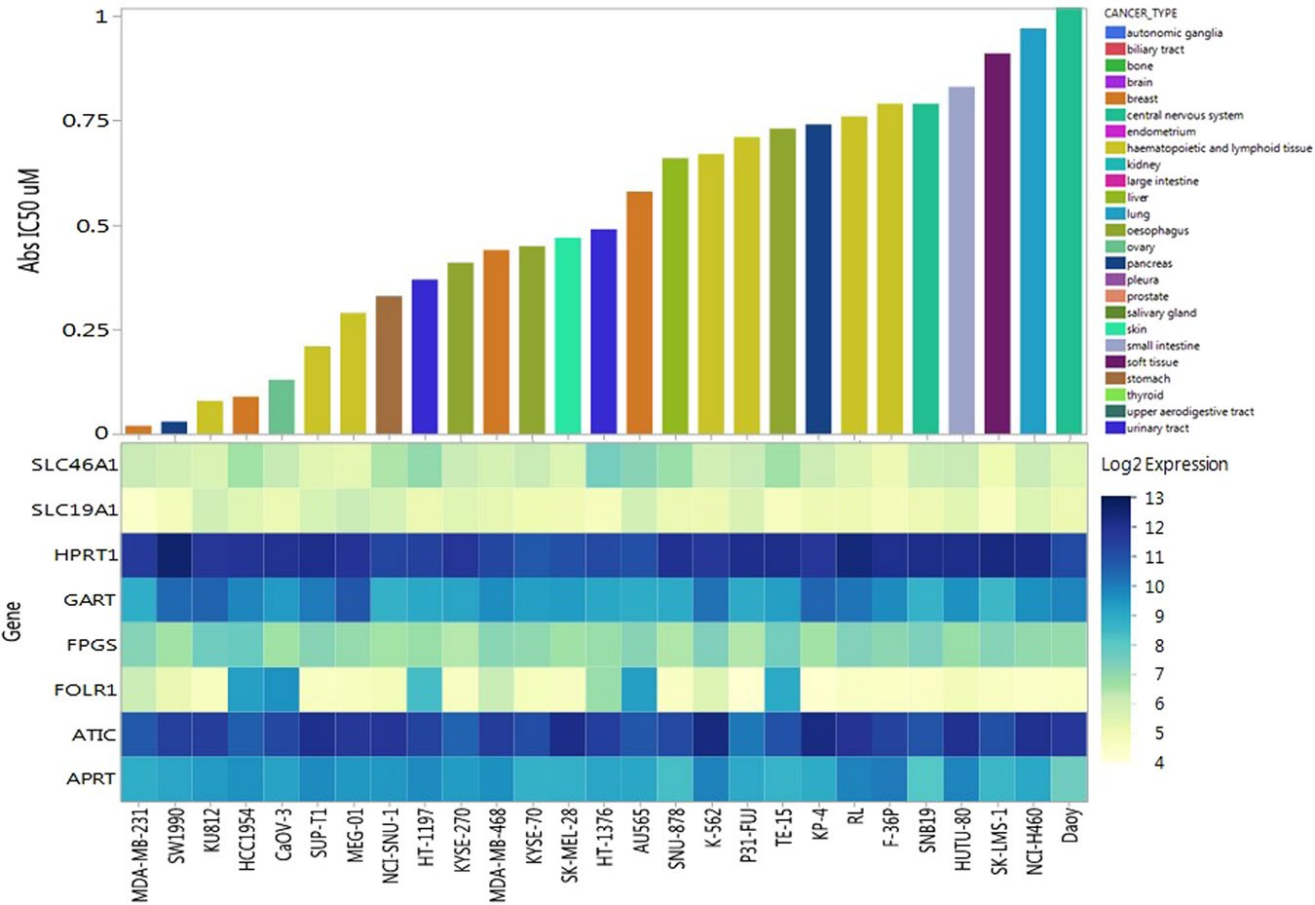
Upper panel: Waterfall plot of anti-proliferation IC50 in μM using Cell Titer Glo after two doubling times for cell lines with IC50s less than 1 μM. Lower panel: Gene expression profile from Affymetrix array for ATIC, GART, APRT, HPRT, FPGS, FOLR1, SLC19A1 and SLC46A1.

Since LSN3213128 was able to compete with the high levels of folate present in standard tissue culture media, it was tested against 298 cell lines at two doubling times using Cell Titer Glo as the read out (Supplemental Figure S1). Cell Titer Glo measures ATP, thus the IC50s represent both the effect on purine production and cell proliferation, which are related. Figure 2 shows only those cell lines with IC50s less than 1,000 nM. Folates are actively taken up in cells by reduced folate carrier (SLC19A1), proton-coupled folate transporter (SLC46A1), and the FOLR1 folate receptor^17^. Once inside the cell, folates are trapped by polyglutamation catalyzed by folylpolyglutamate synthetase (FPGS). While LSN3213128 is not a substrate for FPGS, the cellular EC50 shifts relative to the biochemical IC50 are due to the 10-formylTHF polygultamate having a higher affinity than the 10-formylTHF monoglutamate substrate, which was used in the enzymatic assay. Gene expression data for ATIC, GART, APRT, HPRT, FOLR1, SLC19A1, SLC46A1 & FPGS are shown in Fig. 2, since these genes were anticipated to predict sensitivity. No discernable expression pattern correlated to LSN3213128 was observed. MDA-MB-231 emerged from this screen as the cell line most sensitive to LSN3213128. The MDA-MB-231 cell line is reduced folate carrier (SLC19A1) negative due to epigenetic silencing^18^.

**Figure 3:**
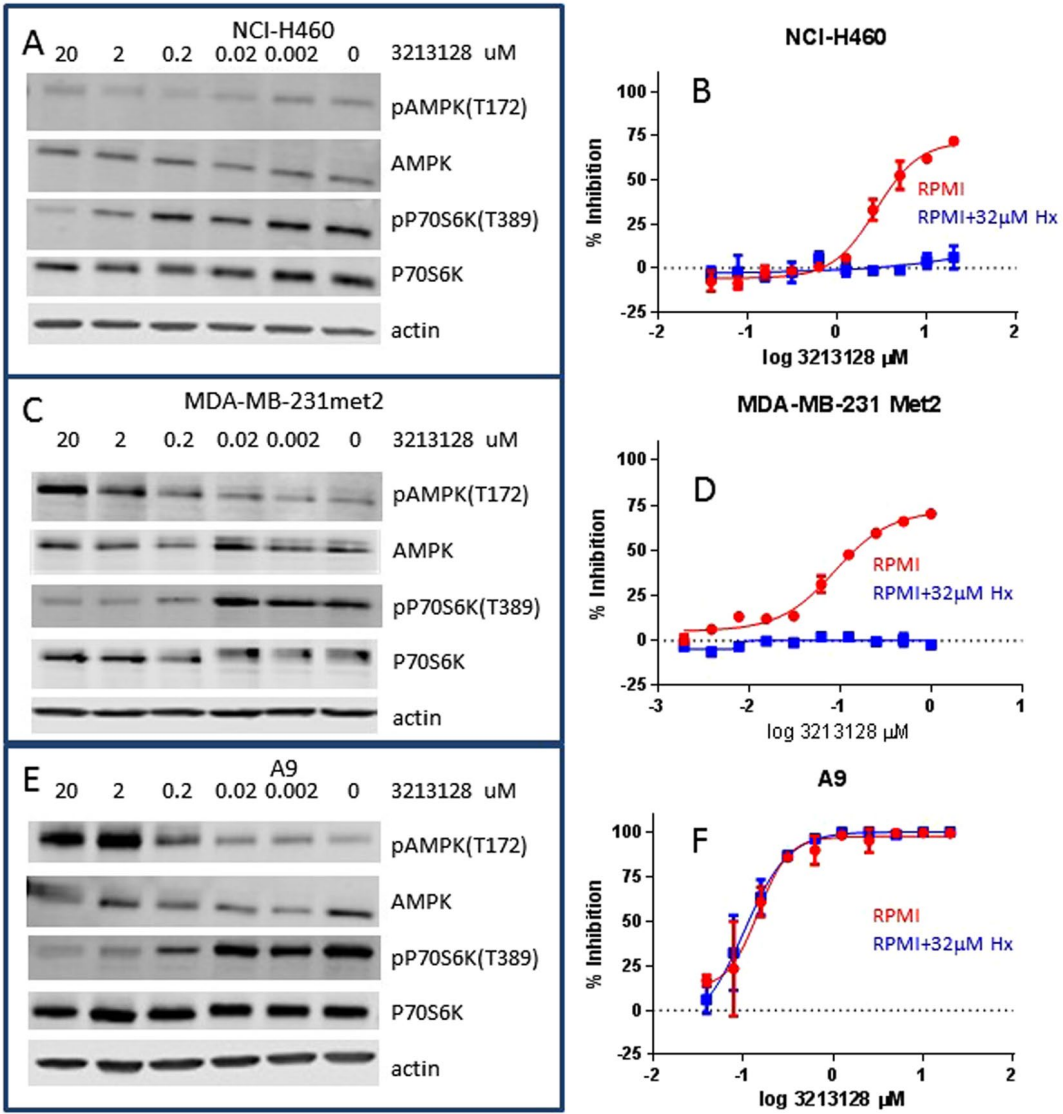
(**A**) The AMPK T172 and P70S6K T389 phosphorylation levels after overnight treatment with 0 to 20 μM LSN3213128 in (**A**) NCI-H460, (**C**) MDA-MB-231met2, and (**E**) A9 using RPMI media, i.e. regular folate conditions. The levels of total actin, AMPKα and P70S6K are also shown. The anti-proliferative effect of LSN3213128 with (blue) and without (red) hypoxanthine in tissue culture are shown as measured using Alamar Blue in (**B**) NCI-H460, (**D**) MDA-MB-231met2, and (**F**) A9. Full images and the croppings used in this figure are shown in Supplemental Figures S4–S13.

Due to mutations in LKB1, NCI-H460 has very low levels of phosphorylated AMPK T172 which are not increased upon treatment with LSN3213128; however, LSN3213128 still inhibits the phosphorylation of P70S6K T389 (Fig. 3A). This inhibition of P70S6K T389 in NCI-H460 occurs near the IC50 of 438 nM for ZMP and Alamar Blue GI50 of 3,470 nM using RPMI media (i.e. Regular Folate in Table 1). Moran suggested phosphorylation of AMPK at T172 is not a clear indicator for activation of AMPK^16^. The anti-proliferative effects of LSN3213128 in NCI-H460 are completely rescued by hypoxanthine supplementation (Fig. 3B). For rescue experiments, 32 μM hypoxanthine was chosen based upon published precedence^3^ which falls within the reported human plasma hypoxanthine levels of 34 ± 10 μM^19^.

The A9 murine cell line is defective in purine salvage (APRT and HPRT deficient)^20^; therefore, A9 is completely dependent on purine biosynthesis. A9 is sensitive to inhibition by LSN3213128 (230 nM IC50 by Alamar Blue in RPMI media) with the highest levels of ZMP in regular RPMI media. No changes in dUMP levels were observed. It should also be noted that GARFT inhibition by lometrexol inhibits ZMP in both A9 and NCI-H460 (data not shown). The high levels of ZMP without dUMP changes in tissue culture demonstrate selectivity of LSN3213128 for AICARFT vs both GARFT and TS. Collectively these studies showing ZMP elevation, AMPK activation and hypoxanthine dependence in tissue culture demonstrate that LSN3213128 inhibits the target, AICARFT, without significant off target activities. Figure 3E shows a similar increase in AMPK T172 phosphorylation for A9 cell line in tissue culture when treated with LSN3213128. A corresponding anti-proliferative effect in A9 treated with LSN3213128 was also observed; however, this effect was not reversed with hypoxanthine supplementation likely due to APRT and HPRT defects in purine salvage (Fig. 3F).

The triple negative breast cancer cell line MDA-MB-231, whose proliferation is sensitive to LSN3213128 (7.6 nM IC50 by CTG and 85 nM by Alamar Blue), grows slowly as a xenograft. Work was transitioned to MDA-MB-231met2 which was found to grow well in murine xenografts. Tissue culture studies in MDA-MB-231met2 dosed with LSN3213128 showed the expected increase in AMPK T172 phosphorylation and the corresponding decrease in the downstream P70S6K T389 phosphorylation (Fig. 3C), similar to the parental cell line (Supplemental Figure S2). The stimulation of AMPK T172 and inhibition of P70S6K T389 in MDA-MB-231 occurs near the IC50 of 89 nM for ZMP and Alamar Blue GI50 of 85 nM using RPMI media (i.e. Regular Folate in Table 1). LSN3213128 produces an anti-proliferative effect in MDA-MB-231met2 with a GI50 of 88 nM in RPMI media which is completely abrogated by supplementation with 32 μM hypoxanthine (Fig. 3D).

LSN3213128 has physical chemical properties, which allow oral dosing for *in vivo* studies. Oral administration of LSN3213128 in mouse at 10 mg/kg resulted in a Cmax of 4567 ± 559 nM (unbound Cmax of 251 ± 31 nM), an AUC of 20222 ± 4518 nM hr and a half-life of 2.4 ± 0.3 h. Intravenous dosing at 1 mg/kg revealed a moderate volume of distribution (779 ± 170 mL/kg) and 24.6 ± 4.6% bioavailability.

**Figure 4:**
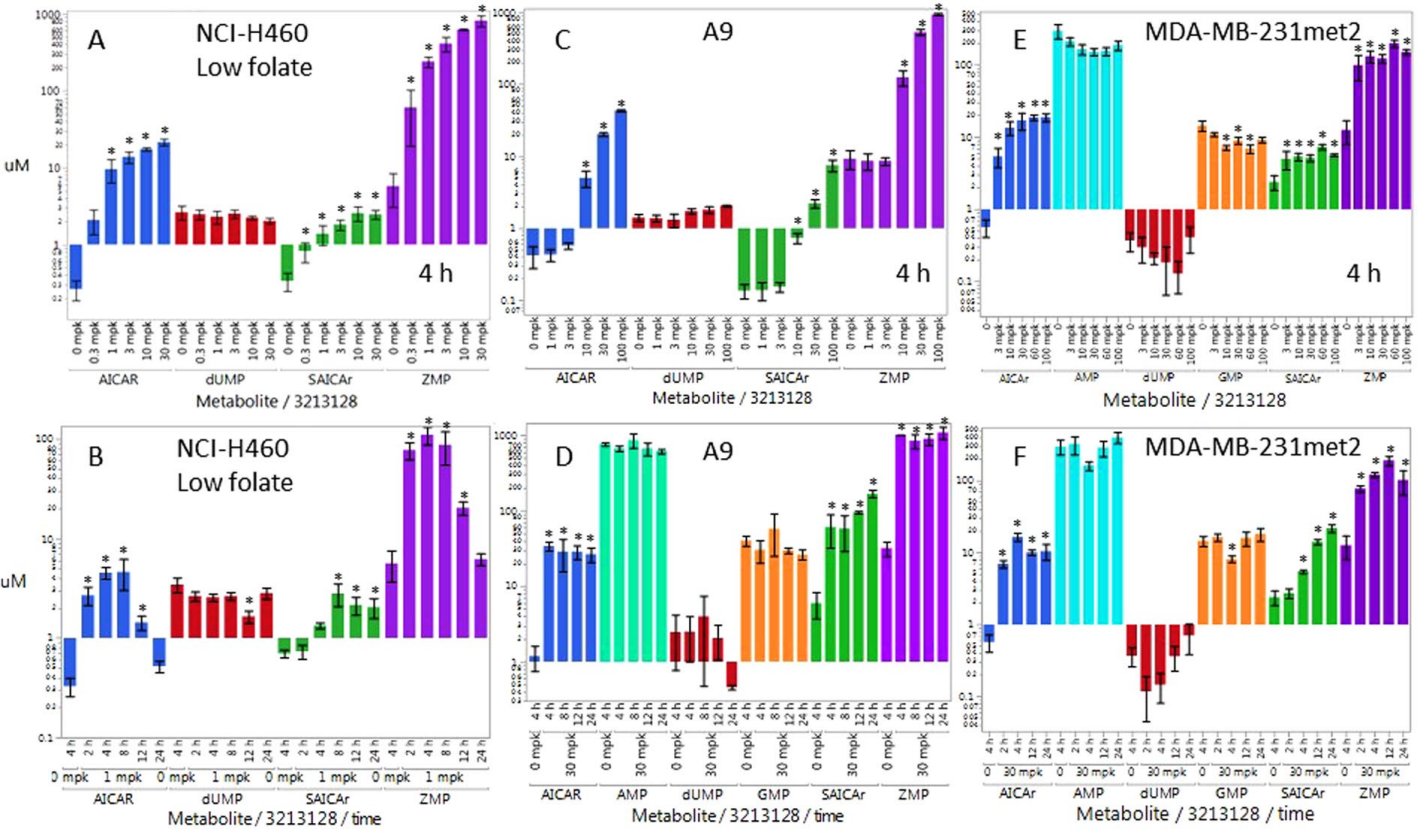
Pharmacodynamic response of ZMP (purple), AICAR (blue), SAICAR (green), dUMP (red), AMP (aqua) & GMP (orange) metabolites in tumors following a single PO dose of LSN3213128 in athymic nude mice. The dose response for the above metabolites following a PO dose of LSN3213128 at 4 hours are shown for (**A**) NCI-H460 on low folate chow, (**C**) A9 on standard chow, (**E**) MDA-MB-231met2 on standard chow. The time course for the above metabolites following a PO dose of LSN3213128 are shown for (**B**) NCI-H460 at 1 mg/kg on low folate chow, (**D**) A9 at 30 mg/kg on standard chow, (**F**) MDA-MB-231met2 at 30 mg/kg on standard chow. A * above the bar indicates a p-value < 0.05 using mean comparisons to vehicle control, Dunnett’s method using JMP 12.1.0.

Initially, a low folate diet was used to reduce the high levels of folate in rodents to within human physiologic range of 15 ± 9 nM^21^. In a representative example, low folate mice had 14 ± 3 nM folate and 25 ± 8 ng/mL B12 whereas normal folate mice had 136 ± 49 nM folate and 30 ± 5 ng/mL B12. The pharmacodynamic dose response 4 h post a PO dose of LSN3213128 in NCI-H460 xenografts grown in nude mice on low folate chow is shown in Fig. 4A. The ZMP response appears to saturate at 1 mg/kg. The ZMP time dependent response following a 1 mg/kg PO dose in low folate chow is shown in Fig. 4B. AICAR levels follow the same pattern as ZMP, as expected. SAICAr levels also rise on treatment with LSN3213128 due to ZMP inhibition of AS; however, the SAICAr signal remains elevated at 24 h while the AICAR levels fall back to base line. The levels of dUMP were unchanged at 4 h even up to 60 mg/kg at 4 h suggesting no inhibition of TS was occurring *in vivo*. Attempts to run efficacy studies in low folate diet nude mice was untenable due to toxicity upon repeated dosing; therefore, *in vivo* work transitioned into standard diet animals.

**Figure 5:**
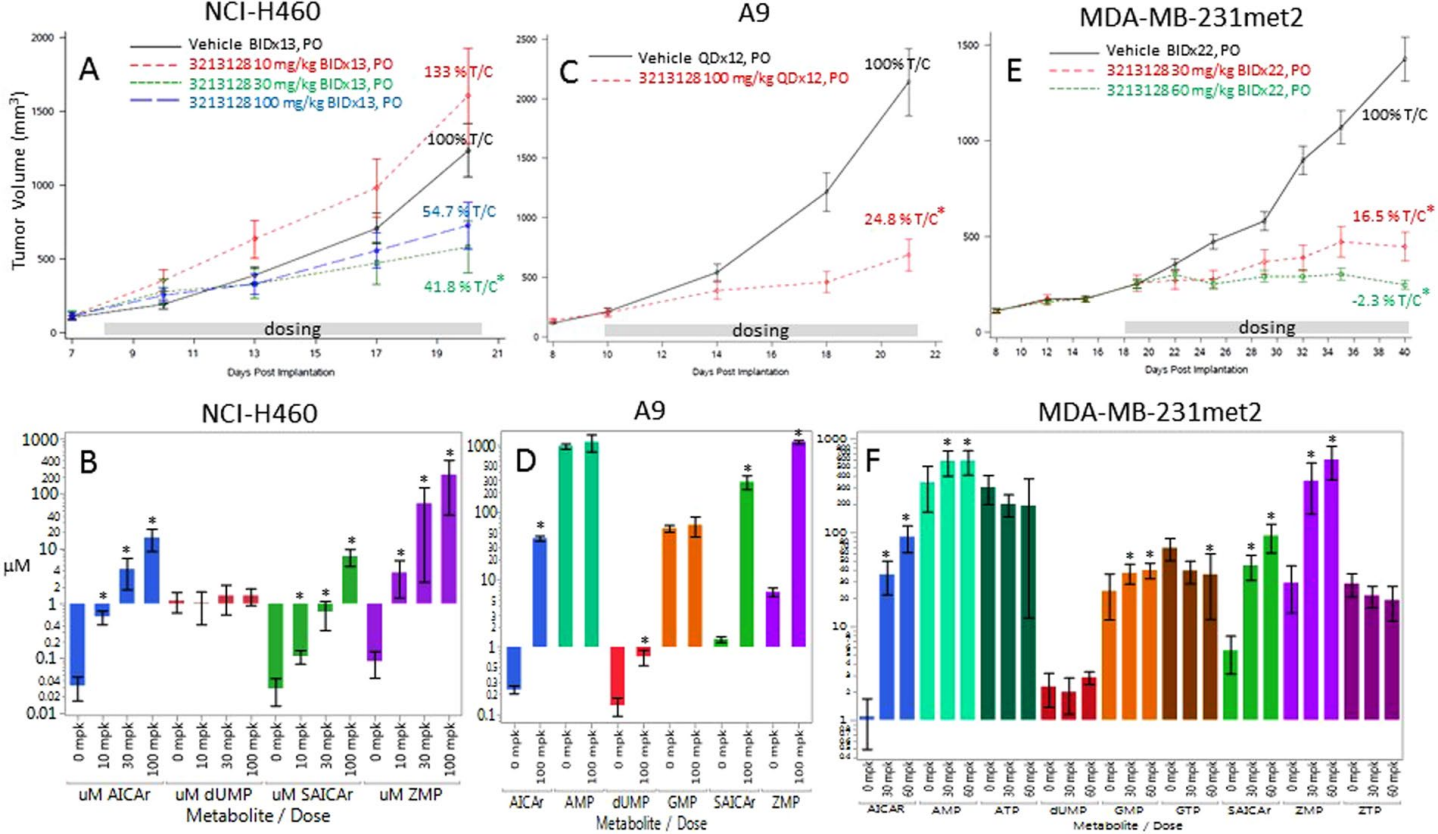
The anti-tumor effect of LSN3213128 dosed orally in mice are shown using the following models: (**A**) NCI-H460 at 10 (red), 30 (green) and 60 (blue) mg/kg BIDx13, (**C**) A9 at 100 (red) mg/kg BIDx12 and (**E**) MDA-MB-231met2 at 30 (red) and 60 (green) mg/kg BIDx22. Vehicle is in black. A * next to the % T/C indicates a p-value < 0.05 compared to vehicle control. The ZMP (purple), AICAR (blue), SAICAR (green), dUMP (red), ATP (dark green),  AMP (aqua), GTP (brown), GMP (orange) & ZTP (plum) metabolite levels following LSN3213128 for the treatment groups above are shown for B) NCI-H460, D) A9 and F) MDA-MB-231met2. A * above the bar indicates a p-value < 0.05 using mean comparisons to vehicle control, Dunnett’s method using JMP 12.1.0.

NCI-H460 tumors were grown in mice on normal folate diet and monitored for efficacy and ZMP levels. LSN3213128 dosed at 30 or 100 mg/kg in mice had anti-proliferative effects on NCI-H460 tumor growth (Fig. 5A). Tumor ZMP levels after 13 days of treatment in normal chow were dose responsive and dramatically elevated (Fig. 5B), but were observed at a significantly higher doses than those required for ZMP elevation in low folate chow (Fig. 4A). ZMP hydrolysis product, AICAR, was readily detected and responsive to LSN3213128 (Fig. 5B). SAICAR levels also rose (Fig. 5B) on treatment, which was due to product inhibition of adenylsuccinate lyase. Levels of dUMP remained constant indicating TS was not inhibited *in vivo*. NCI-H460 was not used to investigate the AMPK activation *in vivo*, because NCI-H460 is LKB1 negative.

The pharmacodynamic response 4 h post a PO dose of LSN3213128 in A9 murine tumors grown in nude mice on standard chow is shown in Fig. 4C. The ZMP response appeared to saturate after 30 mg/kg, similar to what was observed in NCI-H460 on standard chow (Fig. 5B). AICAR and SAICAR dose response in A9 tumors follow the same pattern as ZMP, as expected. ZMP and AICAR levels were sustained after a single 30 mg/kg dose out to 24 h, supporting a QD dosing schedule (Fig. 4D). SAICAr levels rose at 12 and 24 h after a 30 mg/kg PO dose of LSN3213128. The levels of dUMP did not rise at 4 h even up to 100 mg/kg at 4 h, suggesting inhibition of TS was not occurring *in vivo*. AMP and GMP inhibition peak at 24 after a single 30 mg/kg dose, but the effect is modest. Placing the mice on low folate chow prior to implanting the A9 tumor demonstrates that AMP and GMP are indeed responsive to LSN3213128 and that the effect is folate dependent (Supplemental Figure 3).

**Figure 6:**
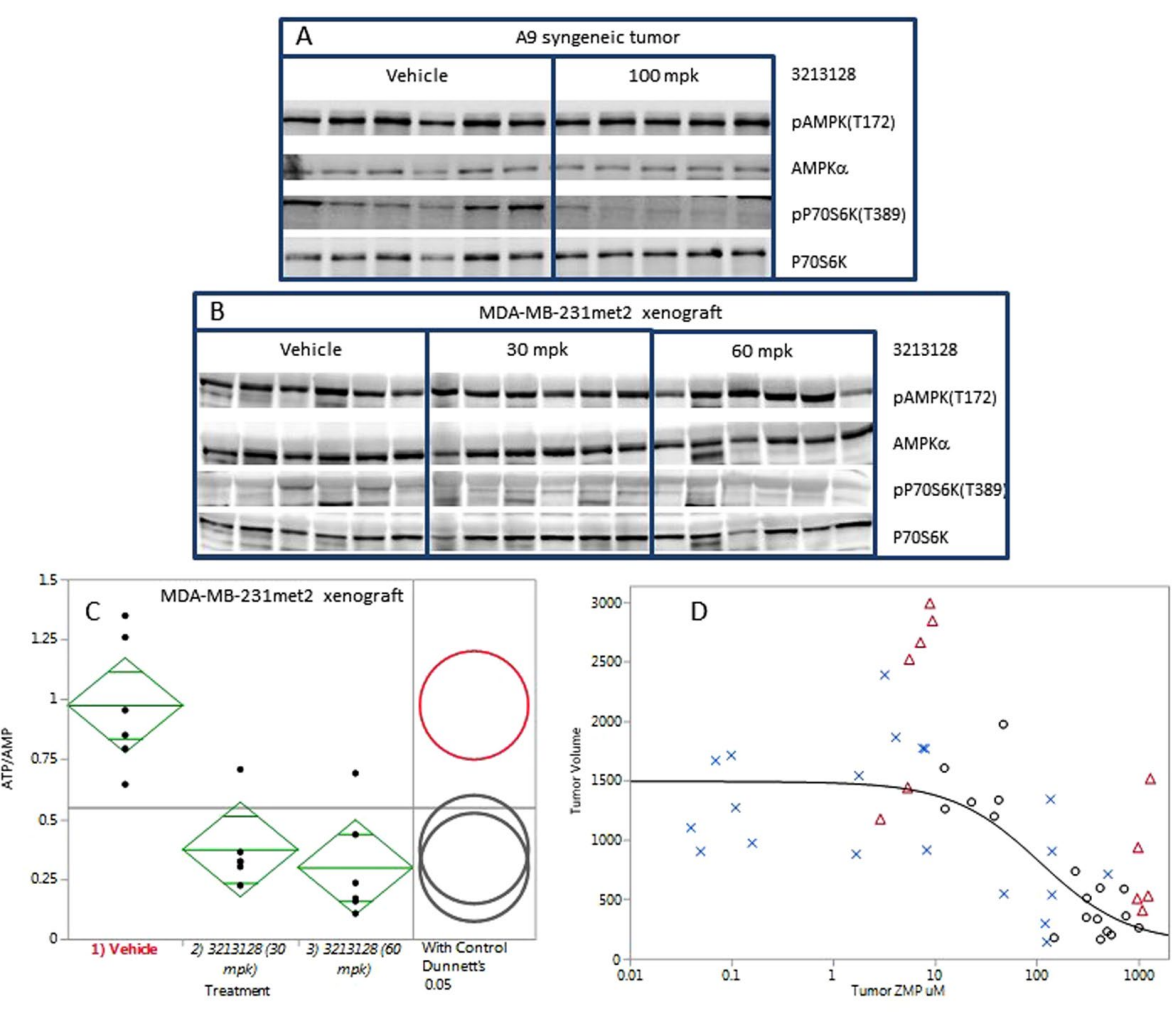
(**A**) The AMPK T172 and P70S6K T389 phosphorylation levels after 100 mg/kg BIDx12 PO dose of LSN3213128 are shown for A9 tumors. The levels of total AMPKα and P70S6K are also shown. (**B**) The AMPK T172 phosphorylation levels after 30 and 60 mg/kg BIDx22 PO dose of LSN3213128 are shown for MDA-MB-231met2 tumors. The levels of total AMPKα are also shown. (**C**) Plot of ATP/AMP ratio after 30 and 60 mg/kg BIDx22 PO dose of LSN3213128 from MDA-MB-231met2 tumors are shown using mean comparisons to vehicle control, Dunnett’s method using JMP 12.1.0. (**D**) A plot shows tumor volume for human large cell lung cancer NCI-H460 (blue X), human triple negative breast cancer MDA-MB231met2 (black circles) and murine A9 tumor (red triangle) vs ZMP levels. The black solid line is a fit of the human MDA-MB-231met2 and NCI-H460 data to a three parameter logistic with an IC_50_ of 108 ± 78 uM, a Max of 1490 ± 120 mm^3^ and a Min of 125 ± 262 mm^3^. Full images and the croppings used in this figure are shown in Supplemental Figures S14–S21.

The lack of change in the purines is possibly due to the tumors salvaging purines. A9 is purine salvage deficient and was not rescued by purine supplementation in tissue culture (Fig. 3F). LSN3213128, when dosed at 100 mg/kg in mice, has anti-proliferative effects on A9 tumor growth (Fig. 5C). After 12 days of dosing, the tumor ZMP levels were dramatically elevated as were AICAR and SAICAR levels (Fig. 5D). AMP or GMP levels showed no changes, and a slight elevation of dUMP was evident in the 100 mg/kg QDx12 dose group (0.72 ± 0.41 uM) relative to vehicle (0.13 ± 0.10 uM). In contrast to tissue culture, AMPK T172 phosphorylation levels *in vivo* were quite high and showed no change on treatment with LSN3213128 (Fig. 6A). The P70S6K T389 phosphorylation signal in A9 was inhibited by LSN3213128.

The pharmacodynamic response 4 h post a PO dose of LSN3213128 in MDA-MB-231met2 xenografts grown in nude mice on standard chow is shown in Fig. 4E. The ZMP response appeared to saturate at 10 mg/kg. AICAR and SAICAR dose response follow the same pattern as ZMP, as expected. ZMP and AICAR levels were sustained after a single 30 mg/kg dose out to 24 h supporting a QD dosing schedule (Fig. 4F). SAICAr levels continued to rise at 12 and 24 h after a 30 mg/kg PO dose of LSN3213128. The levels of dUMP did not rise at 4 h even up to 100 mg/kg at 4 h, suggesting inhibition of TS was not occurring *in vivo*. In MDA-MD-231met2 tumors, AMP and GMP were dose responsive at 4 h post treatment with LSN3213128 but only showed a twofold change in purine levels, as compared to the 15-fold change in ZMP. AMP and GMP inhibition peak at 4–8 h but were lost by 24 h after a single 30 mg/kg dose.

In order to investigate the role of AMPK activation, MDA-MB-231met2 and A9 cell lines were selected based on the activation of AMPK by LSN3213128 in these cell lines (Fig. 3C,E). The assumption based on *in vitro* evidence was that *in vivo* ZMP elevation would lead to AMPK activation and P70S6K activation. LSN3213128 dosed at 30 and 60 mg/kg in mice had anti-proliferative effects on MDA-MB-231 met2 tumor growth (Fig. 5E). After 22 days of dosing, MDA-MB-231 met2 tumor ZMP levels were dramatically elevated as were AICAR and SAICAR (Fig. 5F). Following 22 days of treatment, there were no significant changes in dUMP, AMP, or GMP. In contrast to tissue culture, AMPK T172 phosphorylation levels *in vivo* were quite high and remained elevated even with LSN3213128 treatment (Fig. 6B); furthermore, the P70S6K T389 phosphorylation was weak in MDA-MB-231met2 tumors and showed no change. Changes in phosphorylation of either AMPK or P70S6K cannot be driving tumor growth inhibition. ZMP can be converted to ZTP^22^, leading to the idea that ZTP mis-incorporation into DNA or RNA could be causing the anti-tumor effect. No detectable changes in ZTP were observed (Fig. 5F), thus ZTP is not contributing to tumor growth inhibition. The ATP/AMP ratio is significantly decreased in MDA-MB-231met2 xenografts after 22 days of treatment with LSN3313128 at both 30 and 60 mg/kg doses levels (Fig. 6C).

Overlaying all the individual animal level data for tumor volume versus ZMP levels from Fig. 5 shows a dose response curve for ZMP with an IC50 of 108 ± 78 μM (Fig. 6D) regardless of tumor type. Tumor volume decreases with increasing ZMP; however, in these solid tumors there was no evidence for AMPK activation, either using AMPK phosphorylation at T172 or downstream measures at P70S6K T389. Salvage of purines *in vivo* may explain the absence of purine changes; therefore, we measured levels of hypoxanthine for mouse plasma to be 0.36 ± 0.02 μM (n = 5) which was lower than that reported by Slowiaczek *et al*.^23^. We also quantified hypoxanthine in human plasma, finding the levels were 2.07 ± 0.01 μM (n = 5), well within the 0.45–34 μM range reported in the literature^19,23–29^.

## Discussion

This study describes the characterization of the first potent, AICARFT selective, non-classical, folate competitive inhibitor LSN3213128. Human folate levels in plasma are between 2.5–44.6 nM^21^. Low folate media is traditionally used to test anti-folate compounds, and the ZMP EC50s and Alamar Blue GI50s in low folate are shown in Table 1. Elevated ZMP is observed with LSN3213128 treatment, even under normal tissue culture conditions where the folate is >100 times higher than human plasma concentrations. LSN3213128 is folate competitive as evidenced by the 45-fold shift in potency between low folate and regular media observed with NCI-H460 cell line. The robust elevation of ZMP precludes GARFT inhibition since GARFT activity is required for ZMP production. The compound does not inhibit TS (IC50 > 100 uM) and does not alter the dUMP levels in tissue cultured MDA-MB-231, A9 or NCI-H460 under the conditions used (data not shown).

We characterized LSN3213128 in three cell lines; the triple negative breast cancer cell line MDA-MB-231, the purine salvage deficient murine A9 tumor line, and the LKB1 mutant NCI-H460. Robust elevation of ZMP was observed in all three cell lines. The activation of AMPK was observed in MDA-MB-231 met2 in tissue culture along with a corresponding decrease in the phosphorylation of P70S6K. The results with MDA-MB-231 met2 were consistent with ZMP elevation activating AMPK kinase and inhibiting tumor cell growth; however, hypoxanthine supplementation completely rescues the anti-proliferative effect of LSN3213128. Interestingly the inhibition of phosphorylation of P70S6K T389 was observed in NCI-H460, which has a LKB1 mutation and does not activate AMPK via phosphorylation of T172. The anti-proliferative effect of LSN3213128 in NCI-H460 and rescue by hypoxanthine provides evidence that inhibition of purine biosynthesis contributes to the anti-proliferative activity of LSN3213128. The importance of purine salvage was further emphasized by the failure to rescue the anti-proliferative effect of LSN3213128 in A9 cells, which lack APRT and HPRT and thus cannot salvage purines. The rescue by hypoxanthine in NCI-H460 and MDA-MB-231 in tissue culture demonstrates that the anti-proliferative effect of LSN3213128 is a consequence of purine restriction.

LSN3213128 also inhibits tumor growth in MDA-MB-231met2, A9 and NCI-H460 tumor models. No evidence of alteration in AMPK signaling was observed *in vivo* in either MDA-MB-231 or A9 whereas AMPK activation was observed in tissue culture. Furthermore, we obtained similar efficacy in the salvage deficient murine cell line A9 and the LKB1 mutant NCI-H460 cell line. Tissue culture conditions are significantly different from the *in vivo* model system. The *in vivo* nutrient delivery system has homeostasis maintained in large part by the liver; whereas, in tissue culture high nutrient levels are spiked in and allowed to deplete before refreshing the media. Tissue culture conditions produce a rich energy state with very low phosphorylated AMPK T172 and high-phosphorylated P70S6K T389 (Fig. 3A,C,E). In contrast the *in vivo* environment is energetically challenged as evidenced by the high phosphorylation state of AMPK T172 and the low phosphorylation state of P70S6K T389 (Fig. 6A,B). Figure 6D illustrates the dependence of tumor growth on ZMP levels. These results show that the anti-proliferative activity of AICARFT inhibition via LSN3213128 is correlated with ZMP elevation. AMP and GMP levels remain constant in A9 tumors (Fig. 5D); however, in MDA-MB-231met2, AMP and GMP are significantly elevated and ATP is trending downward, while GTP is significantly lower (Fig. 5F). The lack of reduction in intratumoral AMP and GMP levels was surprising based on published work on Lometrexol^30^ and AG2037^31^, both inhibitors of purine biosynthesis. GARFT inhibition, upstream of ATIC, decreases purine levels after 6 h treatment^32^.

The hypothesis that ZMP elevation activates AMPK is supported by tissue culture data (Fig. 3) but is not supported *in vivo* (Fig. 6A,B). In these solid tumors, energy is already limited and AMPK is maximally activated, shutting down TORC1 signaling. If ZMP is contributing to anti-tumor activity, it cannot be through AMPK activation. At this time the most plausible mechanism is that purine restriction is limiting tumor growth and elevating ZMP. The inhibition of pP70S6k T389 by LSN3213128 in the A9 xenografts (Fig. 6B), which are salvage deficient, supports the purine depletion hypothesis as the depletion of purines leads to inhibition of mTORC1 signaling independent of AMPK^33^. This hypothesis is further supported by an analysis of the ATP/AMP ratio in MDA-MB-231met2 which shows a significant reduction after 22 days of dosing (Fig. 6C).

The translation of activity from tissue culture to *in vivo* tumor models is complicated by plasma folate levels that vary significantly between species: being nearly ~250 nM in rodents, ~25 nM in canine^34^ and 2–45 nM in man^21^. In tissue culture, folate levels in RPMI media is 2,260 nM, which is much higher than plasma levels in any species. To mimic human plasma levels, low folate media is traditionally used in which the folate comes only from the defined fetal bovine serum. Similar to the published work with lometrexol^35^, attempts to lower the folate levels in rodents to human physiologic levels resulted in increased toxicity; therefore, mice were maintained on regular chow. Because LSN3213128 would be dosed in mice on normal diet, cell line screening was performed under standard tissue culture conditions to identify cell lines sensitive to treatment under high folate conditions. In order to compare tissue culture values with tumor levels, an average 4 pL cell volume^36^ was used to calculate cellular ZMP concentrations. In MDA-MB-231met2 cells using regular RPMI, the peak intracellular ZMP levels are about 770 μM while in the salvage deficient A9 cells ZMP is 6,500 μM. In NCI-H460 cells using regular RPMI the peak intracellular ZMP levels are calculated to be about 2,700 μM while in low folate media they are only 540 μM. The levels of ZMP that accumulate upon inhibition by LSN3213128 in tissue culture are dependent on folate, since 10-formylTHF is required to form the precursors for ZMP, i.e. GARFT. While the IC50s are lower in low folate media due to less competition for LSN3213128, the peak ZMP levels are also lower. It is not surprising that *in vivo* in mice on standard chow where folate plasma levels are >10-fold lower than tissue culture that tumor ZMP levels are also lower. As shown in Fig. 6D restricting purine biosynthesis enough to produce >108 μM ZMP is sufficient to reduce tumor growth *in vivo*.

The constant supply of hypoxanthine and additional purines *in vivo* is a complication. Hypoxanthine in human plasma is reported to be as high as 34 ± 10 μM^19^. Purines can be salvaged, which upon conversion to NTP, causing feedback inhibition of purine biosynthesis and blocking ZMP production. The extent to which a human tumor relies on salvage and purine biosynthesis is unknown. In order to establish an estimated human dose required for efficacy one needs to account for both folate and purine levels. Current human dose projection models rely on murine models which have high folate and low hypoxanthine levels that confound the projection of human dose since humans have higher hypoxanthine but lower folate levels.

In summary LSN3213128 is an orally bioavailable, folate competitive, non-classical anti-folate with anti-tumor activity in murine *in vivo* models. The compound is selective for AICARFT relative to other folate dependent enzymes. The anti-proliferative effect is rescued by hypoxanthine in cell lines capable of salvaging purines. Dose projections for humans should account for the differences in folate and hypoxanthine levels between species. LSN3213128 is a potent ATIC inhibitor suitable for clinical development.

## Material and Methods

### Cloning and Enzyme Purification

Human ATIC (accession number: NM_004044.6) cDNA was purchased from Openbiosystem Co. (Cat# MHS1011-62310, Clone ID: 4300570, accession: BC008879). Human TS (accession number: NM_001071.2) cDNA was purchased from Invitrogen (Clone ID: 3138877). Human SHMT (accession number: NM_004169.3) cDNA was purchased from Openbiosystem Co. (Cat# MHS1010-9204609, Clone ID: 4523709, accession: BC038598). Human MTHFD1 (accession number: NM_005956.3) cDNA was purchased from Openbiosystem Co. (Cat# MHS1011-169900, Clone ID: 3508998, accession: BC009806), which contains two mutations and was fixed by PCR-based mutagenesis. The human MTHFD2 (accession number: NM_006636.3) and MTHFD2L (accession number: NM_001144978.1) cDNAs were synthesized at GenScript. The nucleotide sequences encoding full-length human AICARFT, TS, SHMT, MTHFD1, and partial sequences MTHFD2 (32–350) and MTHFD2L (46–347) were inserted into pET21d (Novagen) vector with an N-terminal HIS tag. Bacterial BL21 (DE3) (Novagen) was used as an expression host, and protein purification is described in supplemental material.

### 10 formyl-tetrahydrofolate Substrate Synthesis

Preparation of the 10 formyl-tetrahydrofolate substrate used in the ATIC enzyme assay is a two-step process^37,38^ starting from folinic acid (Sigma CAS 1492-18-8). Final conversion of substrate to 10 formyl-tetrahydrofolate is only stable for same day use.

### Folate dependent Enzyme Assay

Supplemental Table 1 lists the assays used to characterize AICARFT inhibitors. Catalytic activity was assayed under the listed reaction conditions using a liquid chromatograph and either fluorescence or mass spectrometric detection as indicated. Reactions were carried out at RT for the reaction times given. The % response was fit to a four-parameter logistic equation using ACTIVITYBASE 4.0 to determine IC_50_ values.

### Cell culture

Cells lines were acquired from cell banks, see supplemental material, grown using the cell banks recommended culture conditions (Supplemental Table 3). In some experiments noted “low folate” the cells were washed and grown in folate free RPMI-1640 media (Gibco #27016-021) supplemented with 5 ml 1 M Hepes, 5 ml 100 mM Na pyruvate, 2.5 mg/ml glucose and 10% defined FBS (Hyclone #SH30070) per 500 mL media for 5–7 days to deplete intracellular folate levels prior to the start of the experiment. Where noted, cells were maintained in complete “high folate” RPMI-1640 media (ATCC #30-2001) with 10% defined FBS to contrast with the low folate conditions.

The cell line MDA-MB-231 met2 was generated from MDA-MB-231 (ATCC HTB-26) cells co-transfected with reporter plasmids, pGL3 control luciferase (Promega #E1741) and pPUR (Clontech #631601). Puromycin (0.5 µg/ml)-resistant clones were based upon the highest luciferase response. This clone was expanded and injected via tail vein into athymic nude mice. After allowing tumors to establish, the MDA-MB-231met2 clone was isolated from a lung metastasis.

### MS Detection of ZMP in cultured cells

ZMP was detected in cultured cells via mass spec analysis. Cells were plated in 96-well plates at 25,000 cells per well in 100 µL with either high or low folate RPMI media and incubated overnight at 37 °C with 5% CO_2_. Cells were treated with inhibitor compounds for 16 hours, 0.5% DMSO final concentration. Growth media was decanted and cells were lysed for 10 min at room temperature with 50 µL per well of 1x Sure Fire Lysis buffer (Perkin Elmer, SureFire kit component). Aliquots (40 µL) of standard or sample were combined in a deep 96-well plate with 160 µL of internal standard solution containing ^13^C_5_-ZMP (custom synthesis) and ^13^C_5_-AICAr (custom synthesis) at 100 ng/mL in 40 mM ammonium acetate, pH 4. 400 µL of dichloromethane was added to each sample. Samples were sealed, vortexed for 5 minutes, and placed in the refrigerator for at least 30 minutes. The samples were centrifuged at 4 °C for 10 minutes at 16,600 g. 75 µL of the aqueous layer were transferred to a clean 96-well plate and seal prior to analysis.

The LC-MS method utilizes a Shimadzu Prominence 20 A HPLC system connected to an AB Sciex 5500™ or an AB Sciex 6500™ triple quadrupole mass spectrometer. Extracted samples were separated using a Thermo Hypercarb™ Javelin guard column (2.1 × 20 mm, 5 µm) with an injection volume of 15 µL and a flow rate of 1 mL/minute.

ZMP, AICAr and dUMP were detected using positive ion TurboIonSpray® multiple reaction monitoring mode and the data were processed with AB Sciex MultiQuant™. Back-calculated ZMP concentrations were fit to a four-parameter logistic equation using ACTIVITYBASE 4.0 to determine EC_50_ values relative to the maximum observed metabolite levels.

### *In vitro* Proliferation Assays

*In vitro* anti-proliferative activity of LSN3213128 was determined by cell number counting assays against a panel of human cell lines. Cells were cultured using recommended culture conditions unless noted otherwise (Supplemental Table 3). Cells were plated, dosed with compound and incubated at 37 °C for approximately 2 doubling times as determined for each cell line. Cell Titer Glo (Promega) assay was used to measure proliferation according to manufacturer’s recommendations by measuring luminescence with Flexstation3 (Molecular Devices) with 500 ms integration time. The % inhibition for each sample was calculated using 2 μM Staurosporine to define 100% and DMSO to define 0% inhibition. The % response was fit to a four-parameter logistic equation using ACTIVITYBASE 4.0 to determine the absolute IC_50_ values.

### Low folate & high folate *in vitro* proliferation assay

For a limited number of cell lines, compound dosing was performed under high or low folate conditions with cell viability being determined with Alamar Blue rather than an ATP dependent assay. Cells were plated and treated as described above. Proliferation was measured using Alamar Blue Cell Viability Reagent (Invitrogen), and read after approximately 1.5–4 hours incubation at 37 °C on the Envision plate reader (Perkin Elmer) with excitation at 570 nm and emission at 585 nm. The % inhibition for each sample was calculated and fit as described above.

### Antitumor Efficacy in Human Carcinoma Mouse Xenograft Model

All animal studies were performed in accordance with American Association for Laboratory Animal Care institutional guidelines. All Lilly-internal *in vivo* experimental protocols were approved by the Eli Lilly and Company Animal Care and Use Committee. The *in vivo* anticancer activity of LSN3213128 was studied in breast adenocarcinoma cell line MDA-MB-231met2, murine A9 tumor model, or human lung carcinoma NCI-H460 mouse xenograft tumor model. Single dose *in vivo* metabolite studies with NCI-H460 were performed using female athymic nude mice (Harlan) maintained for two weeks on TestDiet 58C3 Folic Acid Def. P.D. w/1% Succinylsulfathiazol (catalog #44840-irradiated) chow, “Low Folate”. *In vivo* studies with MDA-MB-453met2, A9 tumor models and efficacy studies with NCI-H460 were performed using female athymic nude mice (Harlan) on Harlan Teklad Protein Extruded 2920X chow, “Normal Folate”. For all studies the bodyweight was 23–28 g at first measurement and folate (Roche Folate III kit #04476433) and B12 (Roche B12 kit #04745736) levels were monitored prior to implant and at the end of the study using the Roche Elecsys E170 immuno analyzer. The MDA-MB-231met2, NCI-H460 or A9 cells used for implantation were harvested during log phase growth and suspended in serum-free media then diluted 1:1 with Matrigel Matrix (BD 354234). Cells were injected in the right flank with 5 × 10^6^ cells (0.2 mL cell-Matrigel suspension). The LSN3213128 was formulated weekly in 20% HPBCD in Phosphate buffer pH 8 with one molar equivalent NaOH added. The formulated compound was administered at the doses and schedules indicated by oral gavage (0.2 mL). Tumor volume was determined by caliper measurements (mm) and calculated using the formula for an ellipsoid sphere: tumor volume (mm^3^) = length × width^2^/2, where length and width refer to the larger and smaller perpendicular dimensions collected at each measurement. For samples to be analyzed by Western blot or metabolite analysis, the tumors were frozen. The log volume data were analyzed with a two-way repeated measures analysis of variance by time and treatment using the MIXED procedures in SAS software (Version 9.3). The correlation model for the repeated measures was Spatial Power. Treated groups were compared to the control group at each time point. The MIXED procedure was also used separately for each treatment group to calculate adjusted means and standard errors at each time point accounting for the autocorrelation and produced p-values. The *in vivo* effects of AICARFT inhibition on the concentrations of pathway-related analytes were determined by liquid chromatography-mass spectrometry (LC-MS) analysis of tumor xenografts as described in the Supplemental Material.

Delta T/C, % calculation was used. Equations: T = Final tumor volume in treated group; T0 = Baseline tumor volume in treated group (assumed to be same as C0); C = Final tumor volume in control group; C0 = Baseline tumor volume in control group (assumed to be same as T0).

$${\rm{\Delta }}{\rm{T}}/{\rm{C}},\, \% ={\rm{100}}\ast ({\rm{T}}-\mathrm{T0})/({\rm{C}}-\mathrm{C0})$$

### Western analysis

Antibodies were purchased as follows: pAMPK Thr172 (Cell Signaling #2535), p-p70S6 Thr389 (Cell Signaling #9205), total p70S6 (Cell Signaling #2708), total AMPKα (Cell Signaling #2793) and Actin (Sigma #A5441). Frozen tumor samples were lysed with 500 µL ice cold lysis buffer plus Halt protease inhibitor (ThermoFisher #78430) using a Power Gen 125 tissue grinder (Fisher Scientific). Western blots were run as described in the supplemental material.

### Pharmacokinetic Studies

Male CD-1 mice purchased from Charles River Laboratories were dosed with LSN3213128 at 1-mg/kg intravenously or 10-mg/kg orally in 20% 2-Hydroxypropyl-ß-Cyclodextrin in 25 mM Phosphate Buffer with 1 molar equivalent NaOH solution formulation in a cross over design with 3 day wash period in between two arms. Blood samples were obtained through tail clip at 0, 0.08 (IV only), 0.25, 0.75, 2, 4, 8, and 24 hours where approximately 20 µL of blood was filled to capacity into EDTA-coated capillary tubes (Fisher Scientific,) and spotted on to Whatman® DMPK-C DBS collection (GE Healthcare Bio-Sciences). Once spotted, the cards were allowed to dry for 2 hours, placed in Ziploc® bags (S.C. Johnson & Son, Inc.) and shipped to the bioanalytical facility at ambient temperature. Punches (3 mm) from DBS cards were extracted with 180 µL of a 20 ng/mL analog IS (proprietary Lilly compound) in methanol/acetonitrile (1:1, v/v) solution in 96-well plates. Samples were quantitated using LC-MS analysis (AB Sciex® API 4000 triple-quadrupole mass spectrometer (Life Technologies Corporation). PK parameters were calculated for concentration time profiles using Watson LIMS version 7.4 (Thermo Scientific).

The datasets generated during and/or analyzed during the current study are available from the corresponding author on reasonable request.

## Electronic supplementary material

Supplemental information

Supplemental Table 1

Supplemental Table 2

Supplemental Table 3
