# Supplementary material for: Characterization of a novel AICARFT inhibitor which potently elevates ZMP and has anti-tumor activity in murine models
Source: Sci Rep. 2018 Oct 18;8:15458. doi: 10.1038/s41598-018-33453-4 (PMC6193938; doi:10.1038/s41598-018-33453-4)

**Characterization of a novel AICARFT inhibitor which potently elevates ZMP and has anti-tumor activity in murine models.**

Harold B. Brooks^1*^, Timothy I. Meier^1^, Sandaruwan Geeganage^1^, Kevin R. Fales^1^, Kenneth J. Thrasher^1^, Susan A. Konicek^1^, Charles D. Spencer^1^, Stefan Thibodeaux^1^, Robert T. Foreman^1^, Yu-Hua Hui^1^, Kenneth D. Roth^1^, Yue-Wei Qian^1^, Tao Wang^1^, Shuang Luo^1^, Alicia Torrado^1^, Chong Si^1^, James L. Toth^1^, Jefferson R. Mc Cowan^1^, Kwame Frimpong^1^, Matthew R. Lee^1^, Robert D. Dally^1^, Timothy A. Shepherd^1^, Timothy B. Durham^1^, Yong Wang^1^, Zhipei Wu^1^, Philip W. Iversen^1^, and F George Njoroge^1^

^1^Eli Lilly and Company, Indianapolis, Indiana USA 46285

*Corresponding author: brooks_harold@lilly.com

Supplemental Material

Protein purification

The induction of protein expression was carried out in 2xTY media with 1 mM IPTG at 18⁰C overnight. Cell pellets were stored at -80⁰C for subsequent protein purification. Protein purification was conducted at 4⁰C. Frozen cell pellets were lysed by incubation with stirring in 50 ml cold lysis buffer (50 mM Tris-HCl, pH7.5, 300 mM NaCl, 10% Glycerol, 0.1% Triton X-100, 0.5 mg/ml lysozyme, 5U/ml benzonase, 1 mM DTT, 10 mM imidazole, and Roche complete EDTA-free protease inhibitor) per liter cell pellet and sonication. Cell lysates were clarified by centrifugation for 45 min at 23,000g. The supernatant was incubated with Ni-NTA agarose resin (Qiagen) for 3 h, followed by an initial batch wash with 10 resin volume of buffer A (50 mM Tris-HCl, pH7.5, 300 mM NaCl, 10% Glycerol, 1 mM DTT, 10 mM imidazole) containing 0.1% Triton X-100. The resin was then packed onto a column and washed with buffer A. The HIS-tagged protein was eluted with 10-500 mM imidazole gradient in buffer A. Pooled HIS-tagged protein containing fractions were concentrated, loaded onto a HiLoad 26/600 Superdex 200 column (GE Healthcare Biosciences), and eluted with storage buffer (50 mM Tris-HCl, pH7.5, 150 mM NaCl, 1 mM DTT, 10% Glycerol). Fractions containing the HIS-tagged protein were pooled and protein concentration determined by the Bradford assay using BSA as standard. The protein was aliquoted and stored at -80⁰C.

**Cell Lines:** Cell lines of adrenal gland, autonomic ganglia, biliary tract, blood ALL, blood AML, blood CML, blood hodgkin lymphoma, blood lymphoma, blood multiple myeloma, blood NHL, blood RAEB, bone, breast ER+, breast fibroblast, breast fibroblast mixed, breast HER2, breast normal, breast triple negative, cervix, CNS, endometrium, eye, fibroblast normal, kidney ns, kidney renal, large intestine, liver, liver normal, lung adenocarcinoma, lung ns, lung NSCLC, lung NSCLC mixed, lung SCLC, lung squamous, melanoma, melanoma normal, esophagus adenocarcinoma, esophagus ns, esophagus squamous, ovary, pancreas adenocarcinoma, pancreas ductal carcinoma, pancreas ns, pleura mesothelioma, prostate adenocarcinoma, prostate small cell, salivary gland, small intestine, soft tissue, stomach, testis, thyroid, thyroid squamous, umbilical vein endothelial cell normal, upper aerodigestive tract squamous, urinary tract ns, urinary tract transitional cell carcinoma, and vulva squamous origin were obtained from ATCC, CASTCC, CLS, DSMZ, ECACC, HSRRB, ICLC, JCRB, NCI, Riken and SNU cell banks. The murine A9 cell line was acquired from the ATCC (CCL-1.4).

**Metabolite Assays:** Frozen tumor xenograft samples were weighed on an analytical balance, transferred to 2 mL Eppendorf Safe-Lock Tube™ containing a 5 mm steel bead, and placed on dry ice. For every mg of tissue, 10 µL of internal standard solution containing ^13^C_5_-ZMP and ^13^C_5_-AICAr (1000 ng/mL) in ascorbic acid/formic acid/methanol/dichloromethane (0.1:0.1:80:20) was added. Samples were homogenized on a TissueLyser II (Qiagen) for 5 minutes at 15 Hz. Lysates were cleared by centrifugation at 4 ^o^C for 10 min at 16,600 x g. Aliquots of each supernatant (200 µL) were transferred to a deep 96-well plate containing 600 µL dichloromethane and 200 µL 0.1% ascorbic acid solution per well. The plate was sealed, vortexed for 5 minutes, and centrifuged at 4 ^o^C for 5 minutes at 16,600 x g. Transfer 75 µL of the top layer to a clean 96-well plate, and add 75 µL of 40 mM ammonium acetate, pH 4, and seal plate. The LC-MS method utilized a Shimadzu Prominence 20A HPLC system connected to an AB Sciex 5500™ or an AB Sciex 6500™ triple quadrupole mass spectrometer running Analyst® software. Extracted samples were separated using two Thermo Hypercarb™ Javelin guard columns (2.1 x 20 mm, 5 µm) connected in series with an injection volume of 15 µL and a flow rate of 0.75 mL/minute. ZMP, AICAR and dUMP were analyzed in positive ion TurboIonSpray® multiple reaction monitoring mode and the data were processed with AB Sciex MultiQuant™.

Stable-isotope labeling of ^13^C_2_, ^15^N_2_-hypoxanthine, ^13^C_5_-adenosine, d_4_-guanine, ^15^N_5_-GTP, ^13^C_5_-AMP, and other nucleotide metabolites were obtained from Cambridge Isotope Laboratories. LC/ESI/MS/MS analysis of nucleotide metabolites was performed using AB Sciex 6500 mass spectrometer with an ESI probe and interfaced with an LC system. The UPLC system consisted of an Agilent 1290 binary pump, thermostat, TCC, and sampler. Plasma extracted was separated with hypercarb 5μm, 40x2.1 mm Javelin HTS (Thermo Scientific, PN: 35005-022135). Mobile phase A was: water/acetonitrile (95/5): 50 mM ammonium formate, pH 4.0, and mobile phase B was: acetonitrile/methanol (45/50): 50mM ammonium bicarbonate: 0.1% NH_4_OH. Mass spectrometric analyses were performed online using electrospray ionization tandem mass spectrometry in the positive multiple reaction monitoring (MRM) mode. Plasma samples were extracted with methanol/water (80/20) with internal standards. Different nucleotide metabolites were quantified using standard curves and ratios of the peak area of analytes to internal standard.

**Western Blots:** Lysis buffer is 0.01 mg/mL Leupeptin, 0.01 mg/mL Trypsin-Chymotrypsin, 0.01 mg/mL TPCK, 0.1 mg/mL Aprotinin, 60 mM β-Gly PO_4_, 1% Triton-100, 25 mM Tris pH 7.5, 2.5 μM Na_2_Pyrophosphate, 150 mM NaCl, 0.76 mg/mL TAME, 6.2 mg/mL PNPP, 0.8 mg/mL Benzamidine, 0.2 mg/mL Na_3_V0_4_, 0.42 mg/mL NaF, 0.5 mg/mL PMSF or PEFA, 1 mM DTT, 15 mM EDTA pH 8.0, 5 mM EGTA pH 8.0, 1 μM Okadaic acid, and 1 μM microcystine. RNA and DNA were degraded by addition of Benzonase (Novagen) with a 30 min incubation on ice. Lysates were cleared by centrifugation at 4 ^o^C for 10 min at 16,000 x g. Protein content was measured using Pierce BCA protein assay kit (Thermo Scientific). Twenty ug of total protein was resolved on a 4-20% Tris HCl Criterion XT precast gel (BioRad) at 100 V for 90 minutes. Transfer electrophoresed proteins to a 0.2 μm nitrocellulose membrane using Trans Blot Turbo System (BioRad). Block membranes for 1 hr at RT in Odyssey blocking buffer (LiCor). Probe overnight 4°C for primary antibodies. Wash blots 3 x 5 min in 20 mL PBS-0.1% Tween-20. Apply appropriate secondary antibody for 1-3 hours RT. Wash blots 3 x 5 min in 20 mL PBS-0.1% Tween20 and develop signal on Odyssey CLx Infrared Imaging System (LiCor).

For the in vivo study with MDA-MB-231met2 (Figure 6A) 40 ug of total protein was resolved on a 10% Tris HCl Criterion XT precast gel (BioRad) at 100 V for 90 minutes. Transfer electrophoresed proteins to a 0.2 μm nitrocellulose membrane using Trans Blot Turbo System (BioRad). Block membranes for 1 hr at RT in 5% skim milk. Probe overnight 4°C for primary antibodies. Blots were washed 3-5 min in 20 mL TBS-0.1% Tween-20. Apply appropriate secondary antibody for 1-3 hours RT. Wash blots 3 x 5 min in 20 mL TBS-0.1% Tween20 and develop signal using Super Signal Western Femto kit (Thermo) on Image Reader LAS-4000 (Fuji Film Life Science).

Supplemental Figure S1: Waterfall plot of anti-proliferation IC50 in μM using Cell Titer Glo after two doubling times for 298 cell lines organized by tissue types with a few representative cell lines named.


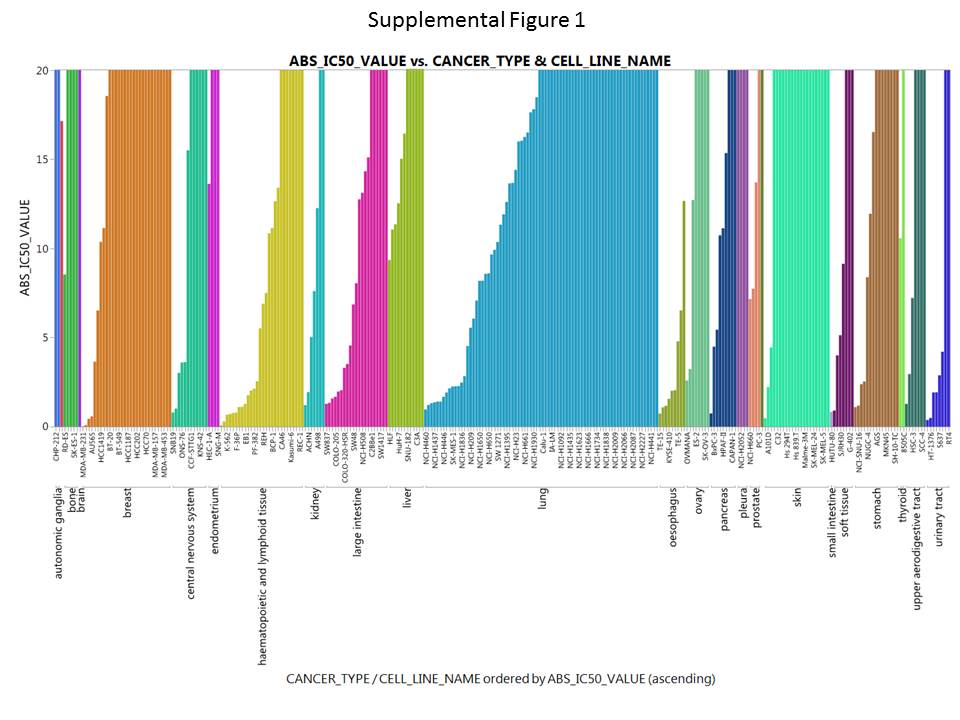


Supplemental Figure S2. Left Panel: The AMPK T172 and P70S6K T389 phosphorylation levels after overnight treatment with 0 to 20 uM LSN3213128 in MDA-MB-231. The levels of total actin, AMPKα and P70S6K are also shown. Right Panel: The anti-proliferative effect of LSN3213128 with (blue) and without (red) hypoxanthine in tissue culture are shown as measured using Alamar Blue in MDA-MB-231.


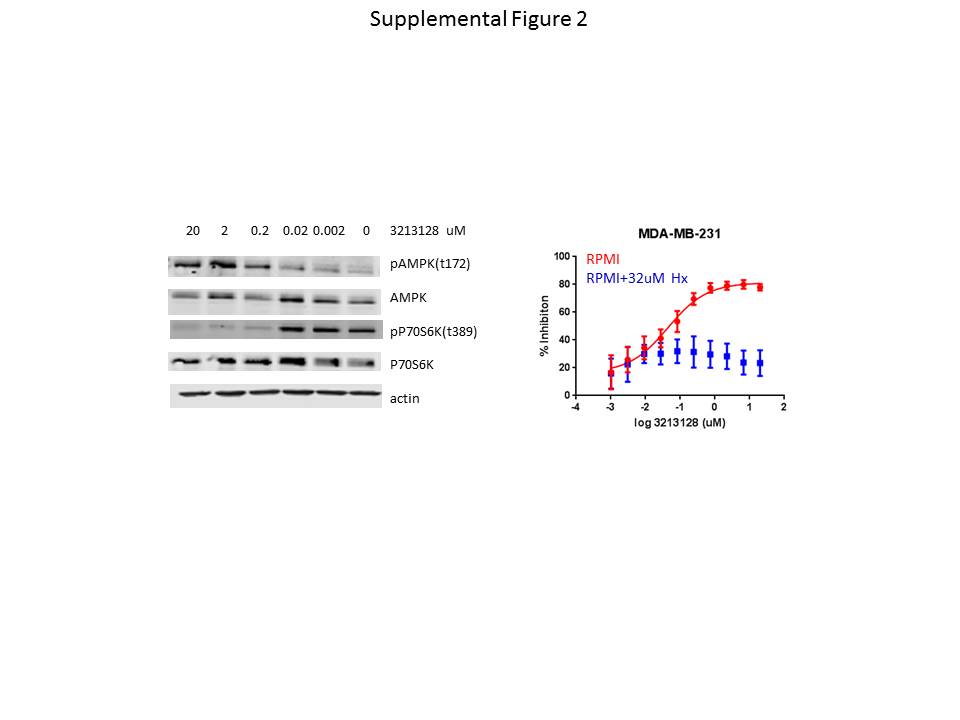


Supplemental Figure S3. The ZMP (purple), AICAR (blue), SAICAR (green), dUMP (red), AMP (aqua) & GMP (orange) metabolite levels following LSN3213128 for the treatment groups above are shown for A9 tumors on low folate chow. A * above the bar indicates a p-value < 0.05 using mean comparisons to vehicle control, Dunnett’s method using JMP 12.1.0.


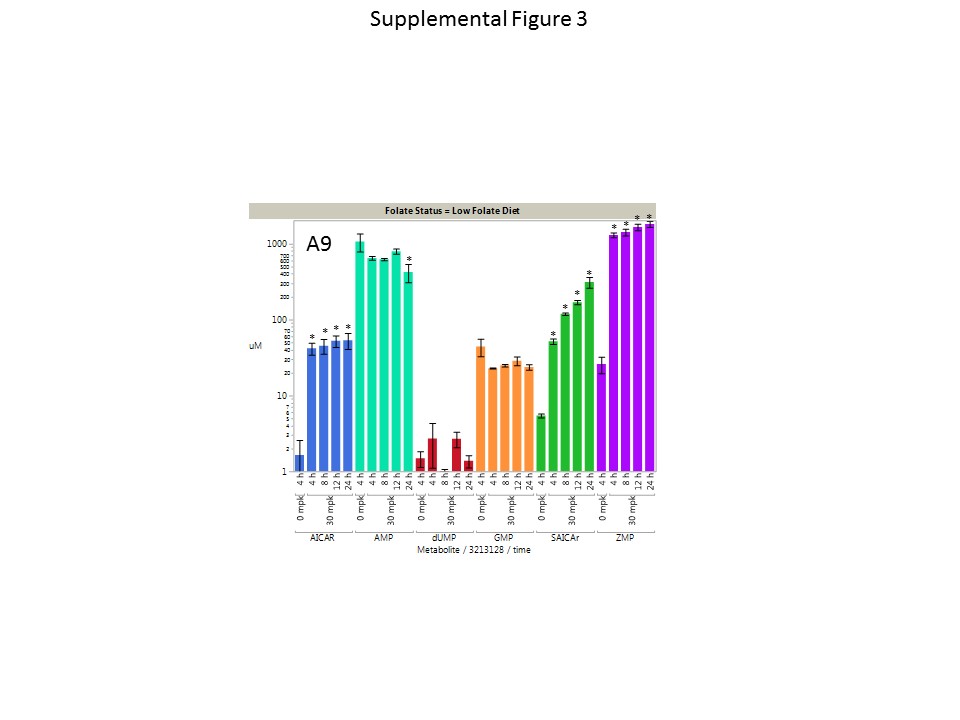


Supplemental Figure S4. Uncropped image for pAMPK Thr172 for Figure 3 panels A and E. The blue boxes show the lanes displayed in Figure 3 panel A & E.


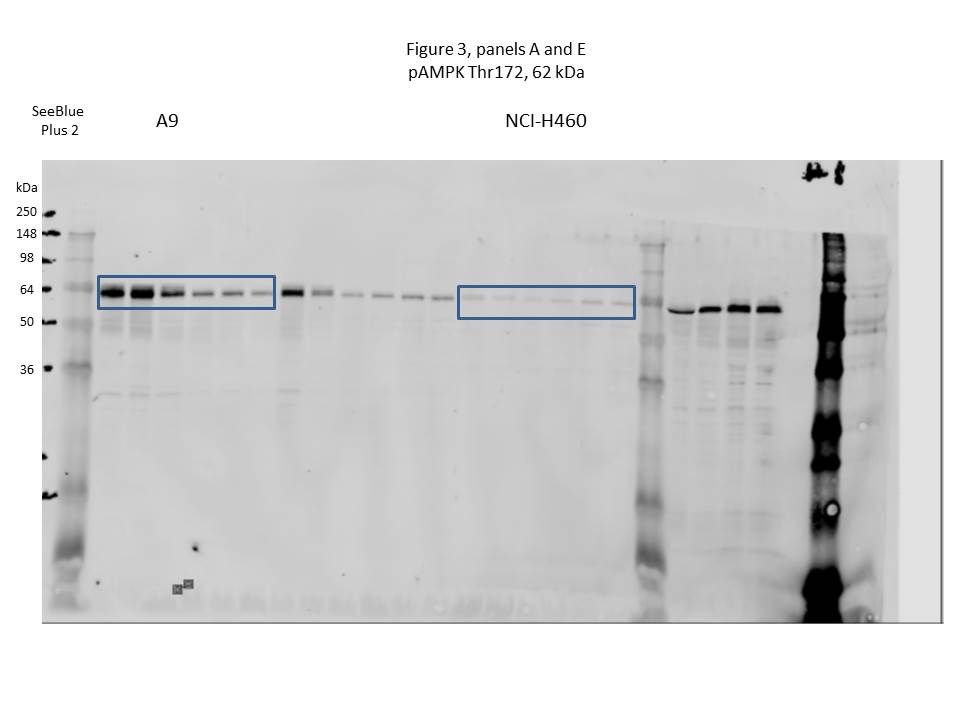


Supplemental Figure S5. Uncropped image for AMPKα for Figure 3 panels A and E. The blue boxes show the lanes displayed in Figure 3 panel A & E.


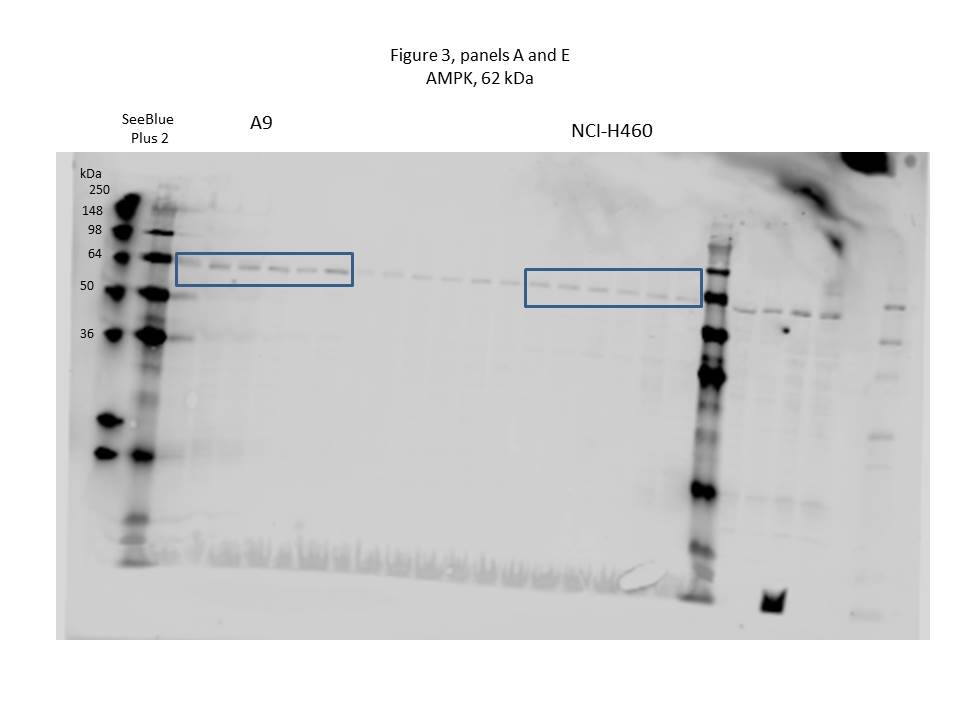


Supplemental Figure S6. Uncropped image for pP70S6K Thr389 for Figure 3 panels A and E. The blue boxes show the lanes displayed in Figure 3 panel A & E.


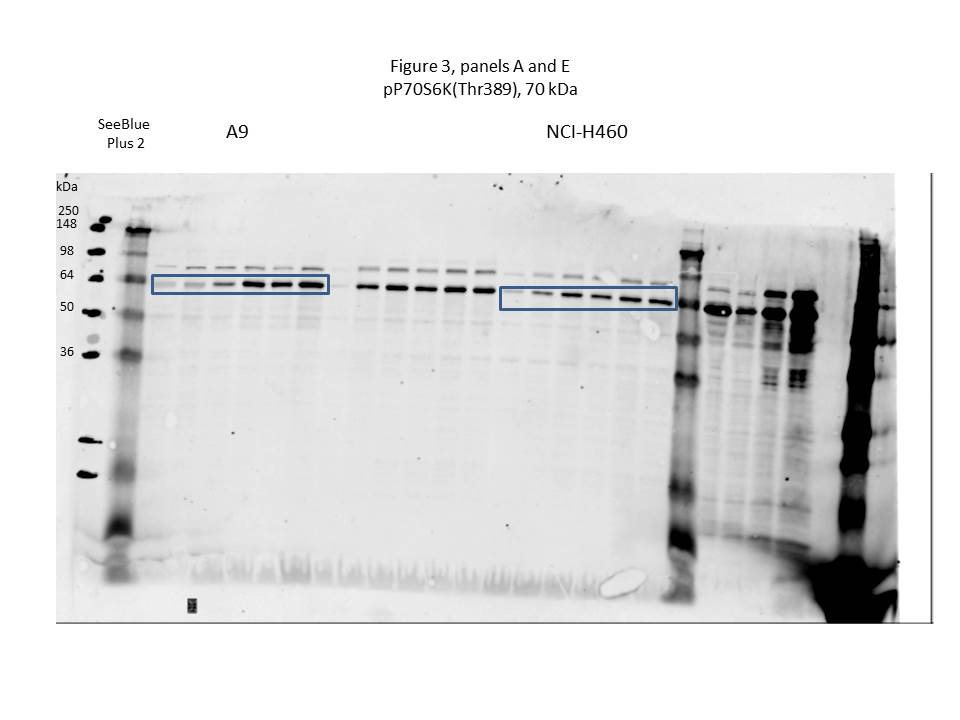


Supplemental Figure S7. Uncropped image for P70S6K for Figure 3 panels A and E. The blue boxes show the lanes displayed in Figure 3 panel A & E.


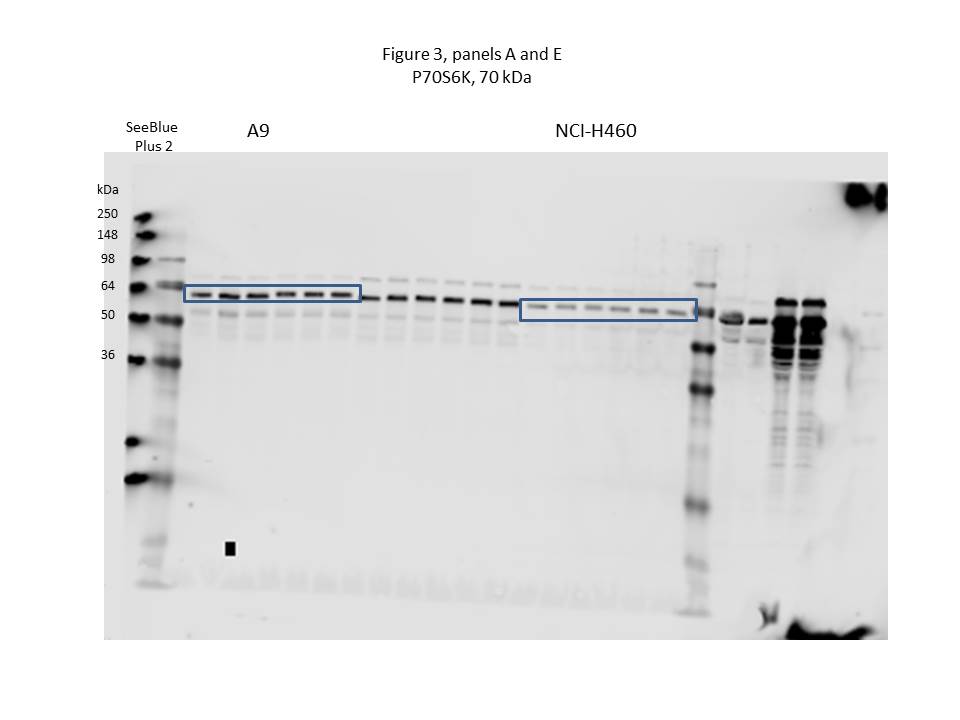


Supplemental Figure S8. Uncropped image for actin for Figure 3 panels A and E. The blue boxes show the lanes displayed in Figure 3 panel A & E.


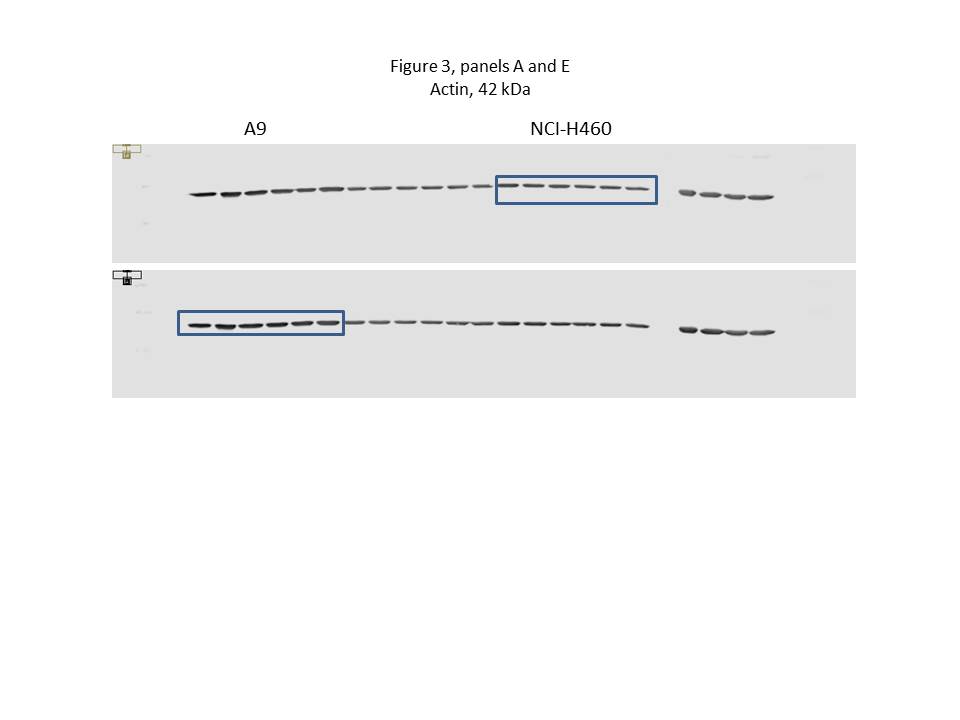


Supplemental Figure S9. Uncropped image for pAMPK Thr172 for Figure 3 panel C and Supplemental Figure S2. The blue boxes show the lanes displayed in Figure 3 panel C and Supplemental Figure S2.


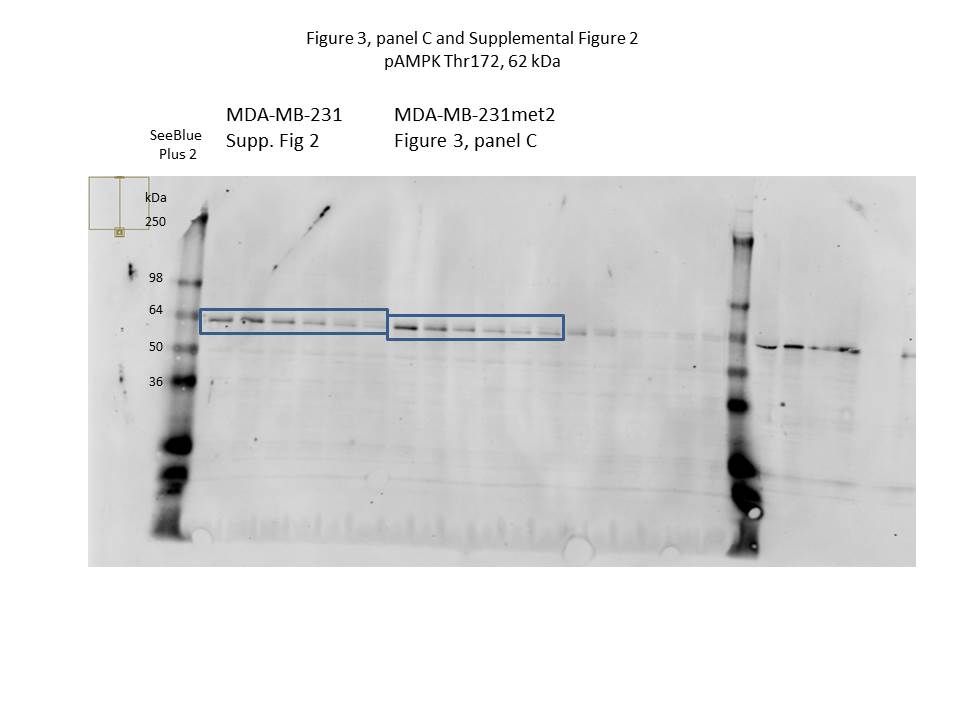


Supplemental Figure S10. Uncropped image for AMPKα for Figure 3 panel C and Supplemental Figure S2. The blue boxes show the lanes displayed in Figure 3 panel C and Supplemental Figure S2.


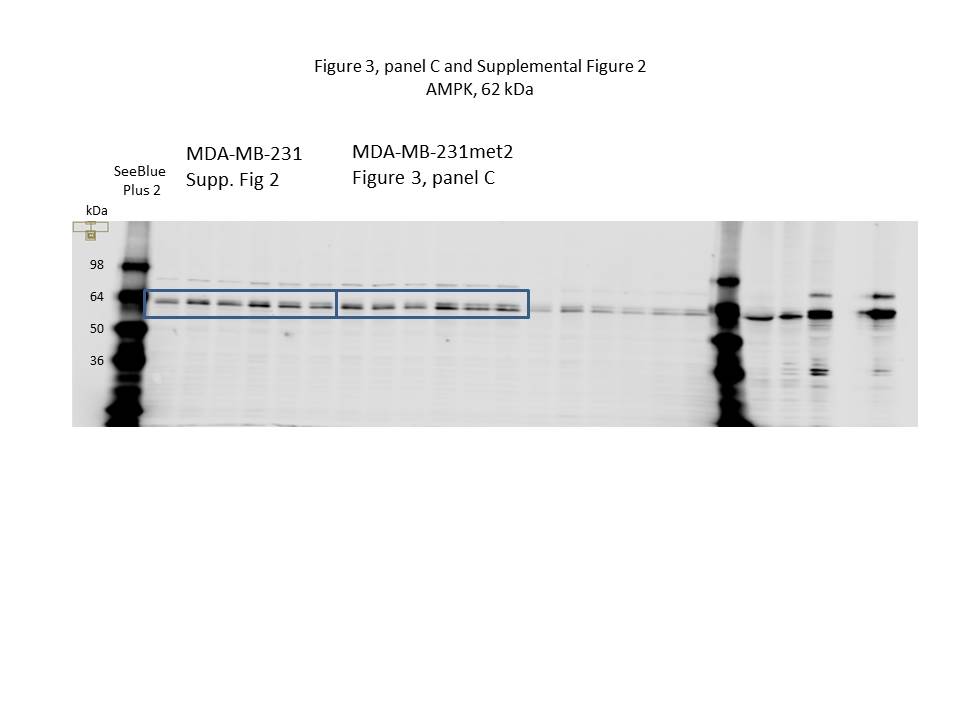


Supplemental Figure S11. Uncropped image for pP70S6K Thr389 for Figure 3 panels C and Supplemental Figure S2. The blue boxes show the lanes displayed in Figure 3 panel C and Supplemental Figure S2.


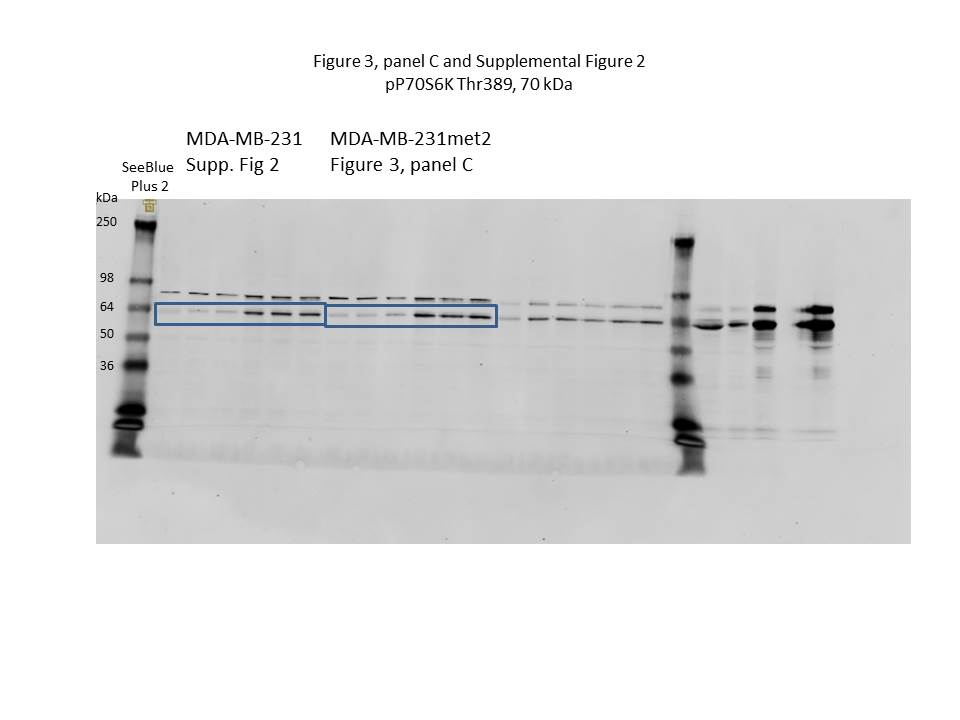


Supplemental Figure S12. Uncropped image for P70S6K for Figure 3 panel C and Supplemental Figure S2. The blue boxes show the lanes displayed in Figure 3 panel C and Supplemental Figure S2.


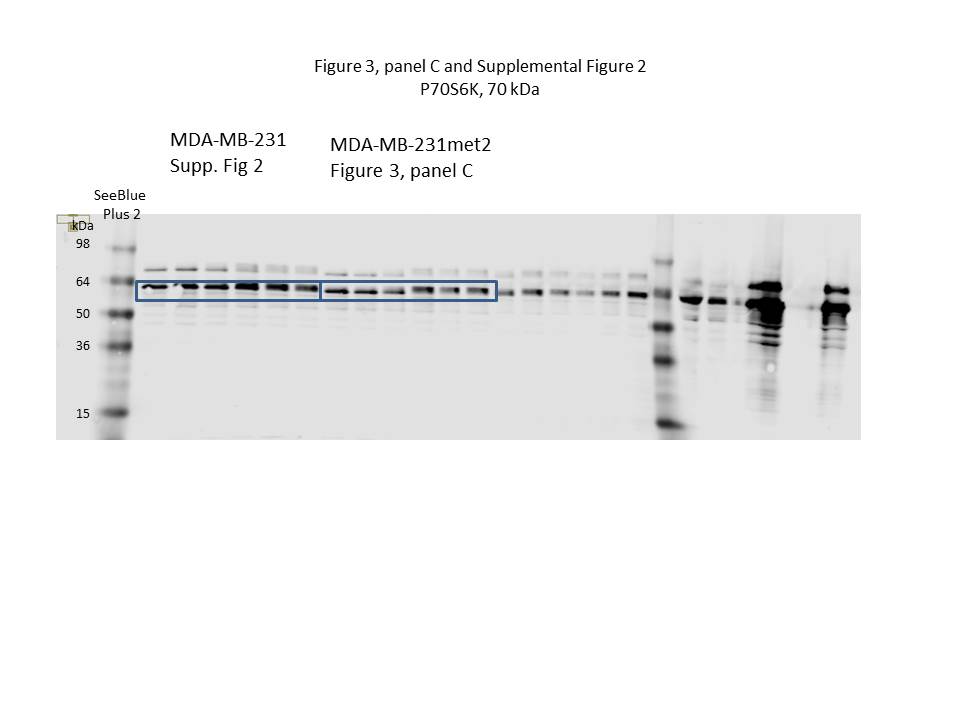


Supplemental Figure S13. Uncropped image for actin for Figure 3 panel C and Supplemental Figure S2. The blue boxes show the lanes displayed in Figure 3 panel C and Supplemental Figure S2.


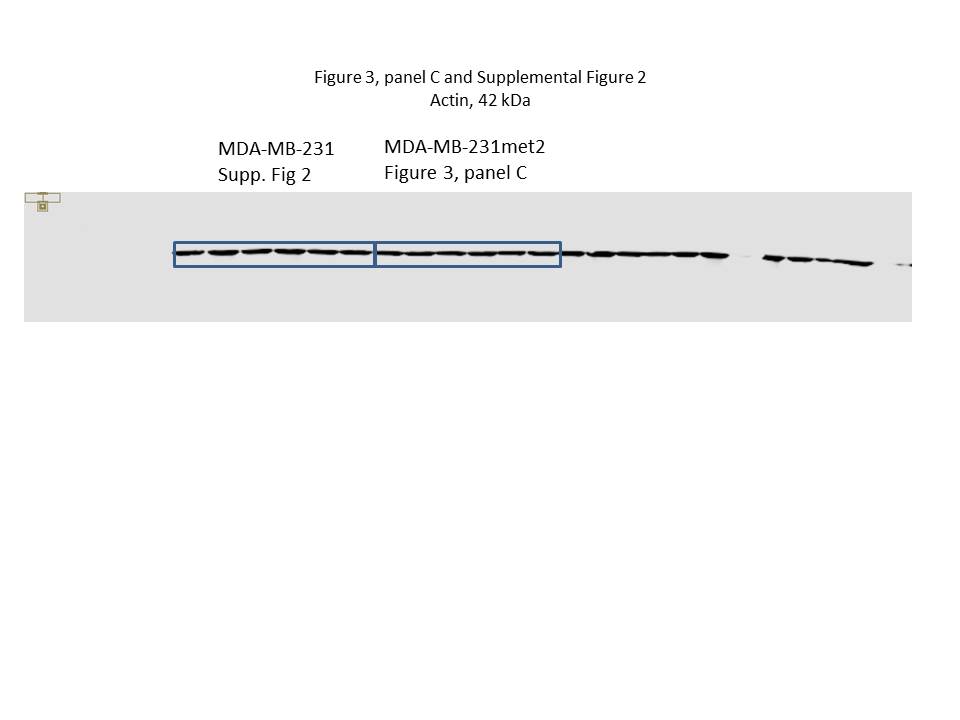


Supplemental Figure S14. Uncropped image for pAMPK T172 for Figure 6 panels A at two exposures. The blue box shows the lanes displayed in Figure 6A.


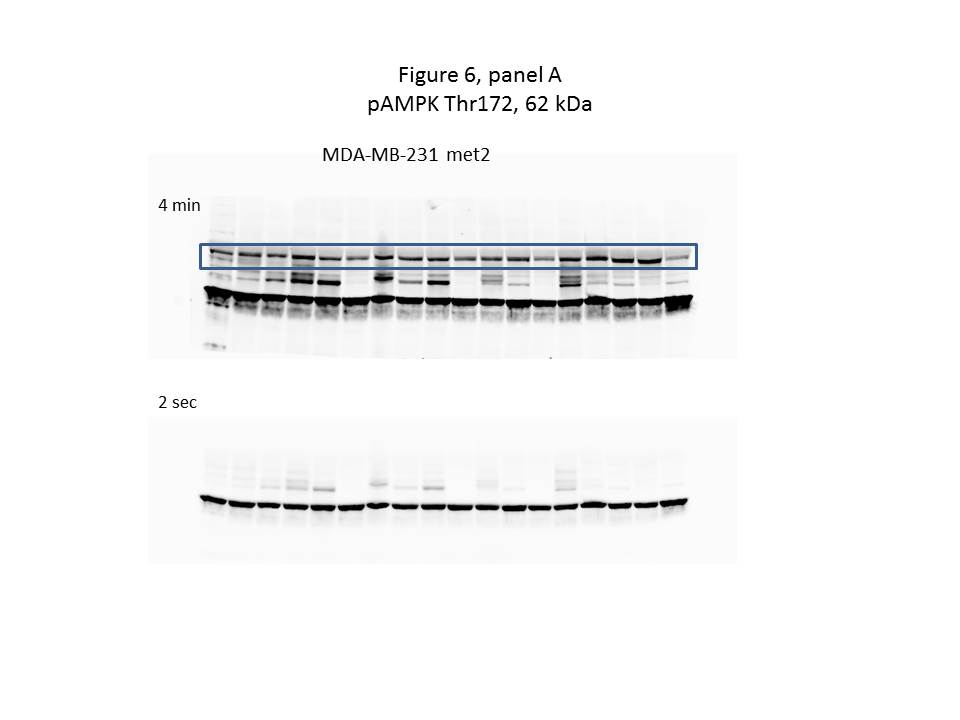


Supplemental Figure S15. Uncropped image for AMPKα for Figure 6 panels A at two exposures. The blue box shows the lanes displayed in Figure 6A.


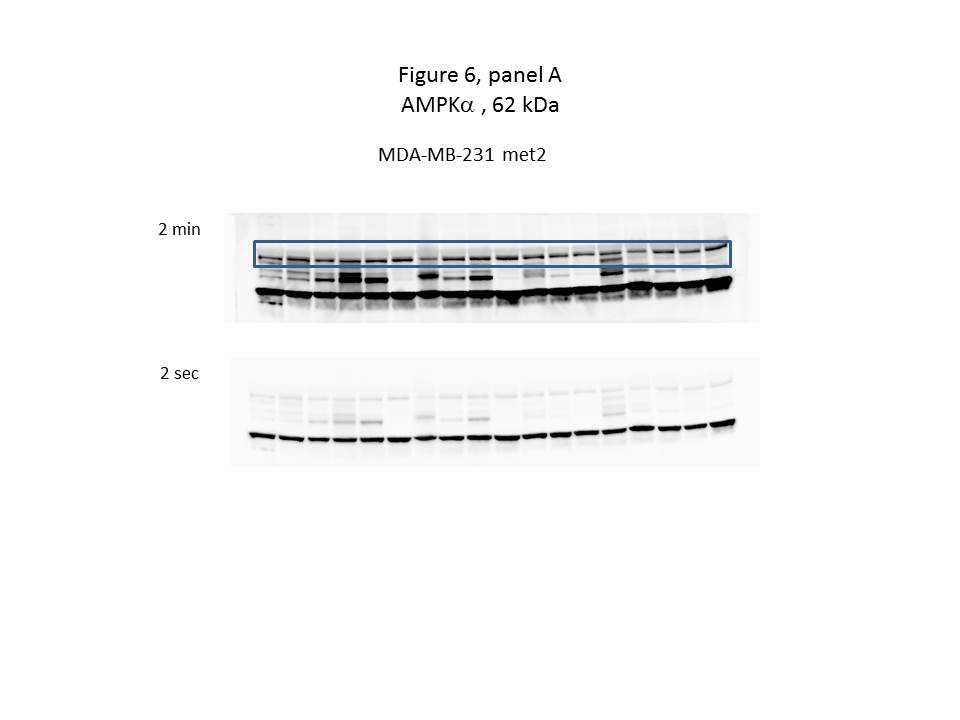


Supplemental Figure S16. Uncropped image for pP70S6K Thr389 for Figure 6 panel A at two exposures. The blue boxes show the lanes displayed in Figure 6A, noting that two crops were used to align the lanes with other images.


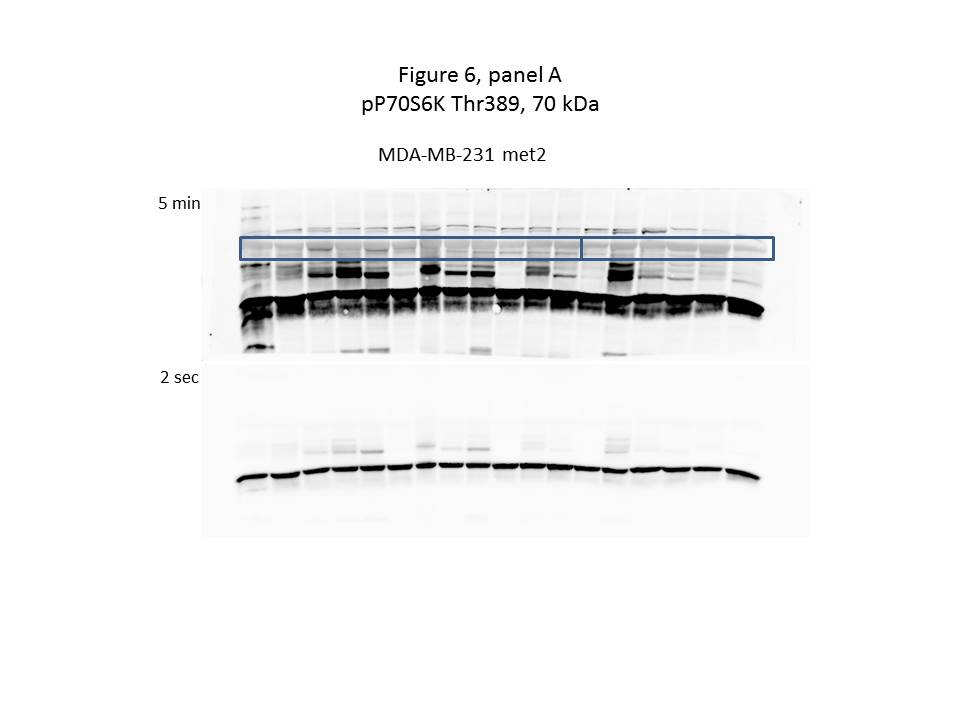


Supplemental Figure S17. Uncropped image for P70S6K for Figure 6 panels A at two exposures. The blue box shows the lanes displayed in Figure 6A.


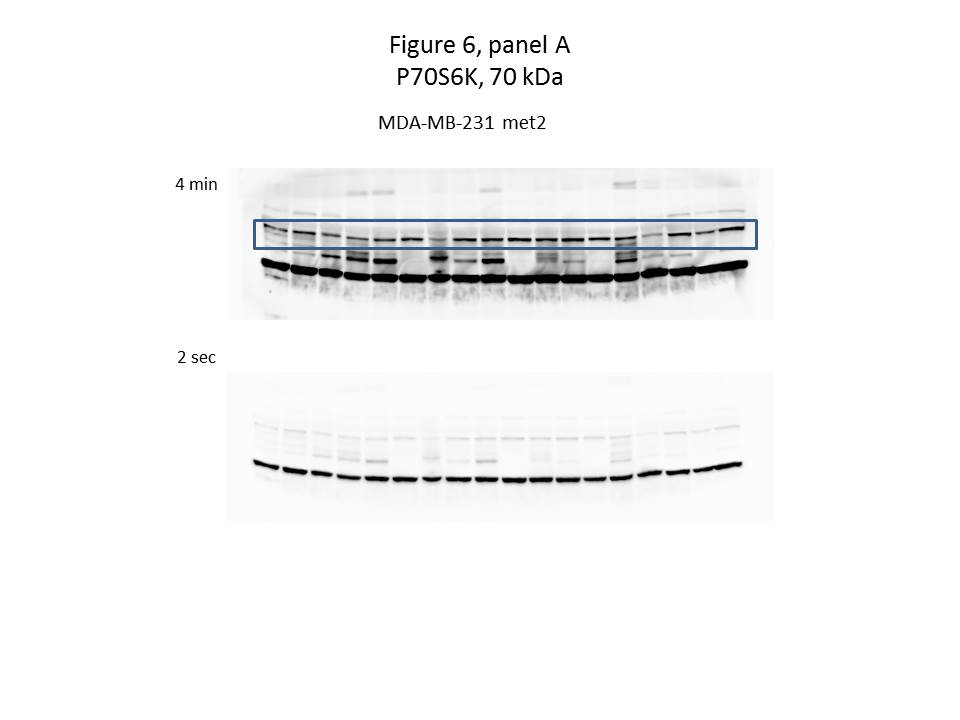


Supplemental Figure S18. Uncropped image for pAMPK Thr172 for Figure 6 panels B at two contrasts. See blue plus 2 markers from ThermoFisher were used to determine molecular weight. The blue box shows the lanes displayed in Figure 6B.


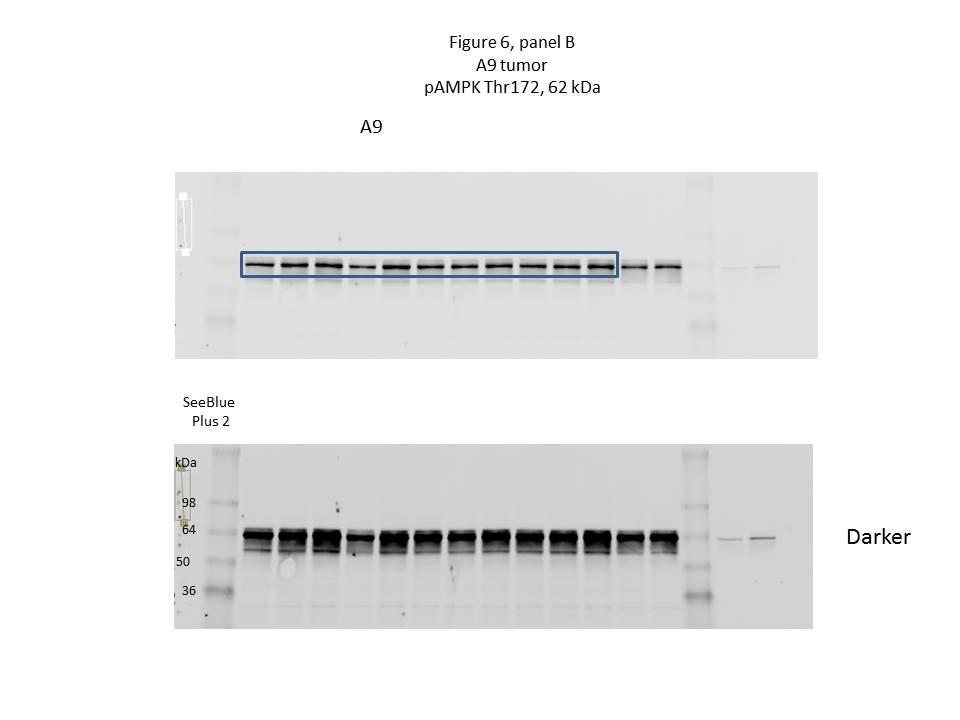


Supplemental Figure S19. Uncropped image for AMPKα for Figure 6 panels B. See blue plus 2 markers from ThermoFisher were used to determine molecular weight. The blue box shows the lanes displayed in Figure 6B.


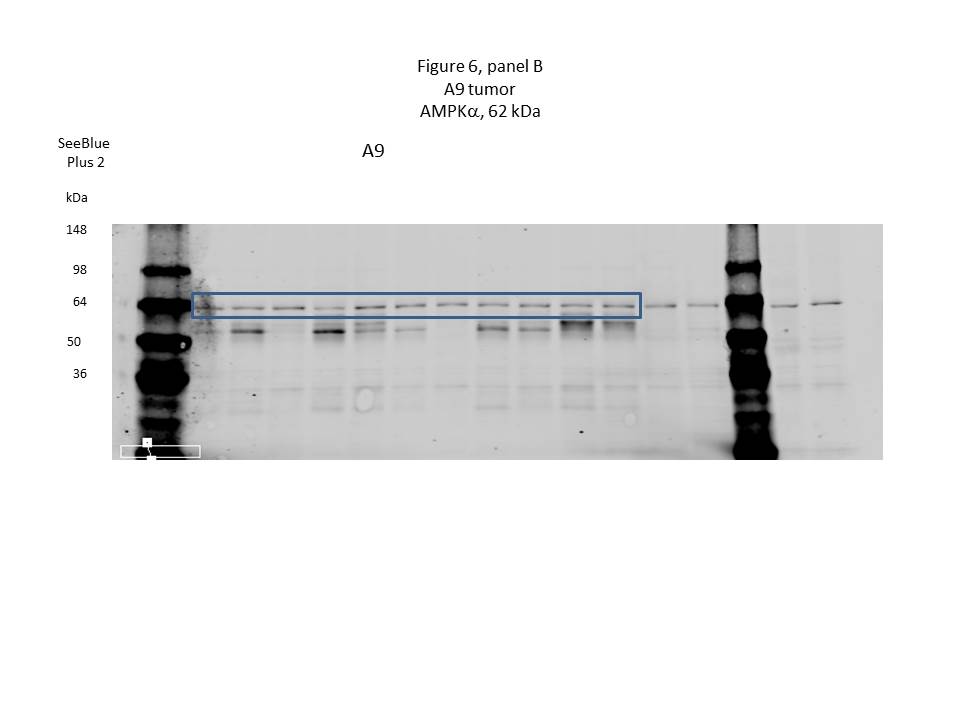


Supplemental Figure S20. Uncropped image for pP70S6K T389 for Figure 6 panels B. See blue plus 2 markers from ThermoFisher were used to determine molecular weight. The blue box shows the lanes displayed in Figure 6B.


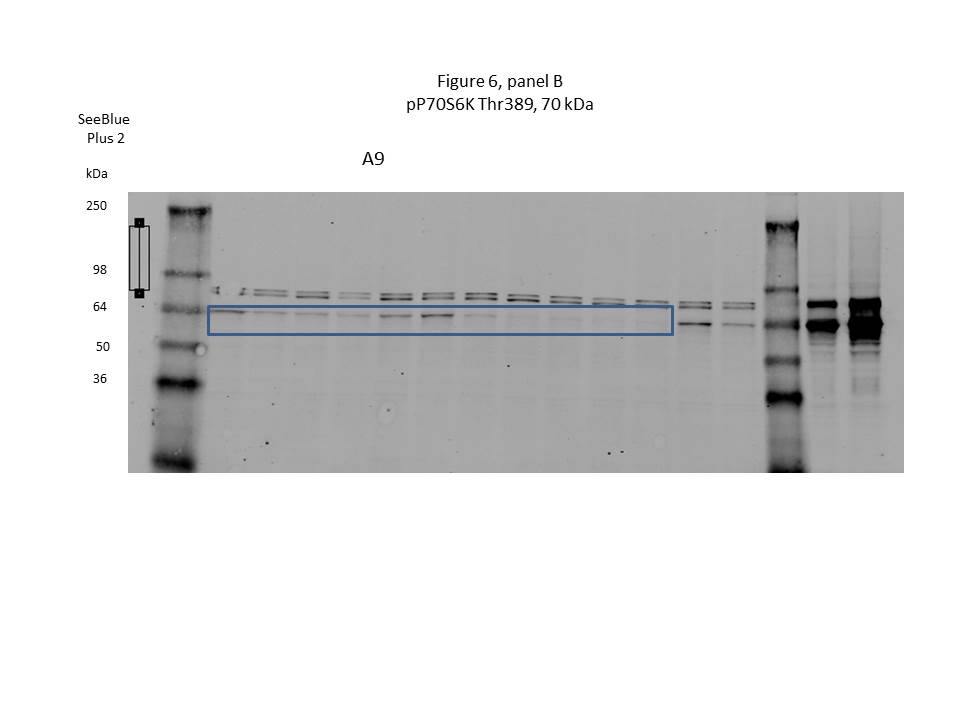


Supplemental Figure S21. Uncropped image for P70S6K for Figure 6 panels B. See blue plus 2 markers from ThermoFisher were used to determine molecular weight. The blue box shows the lanes displayed in Figure 6B.


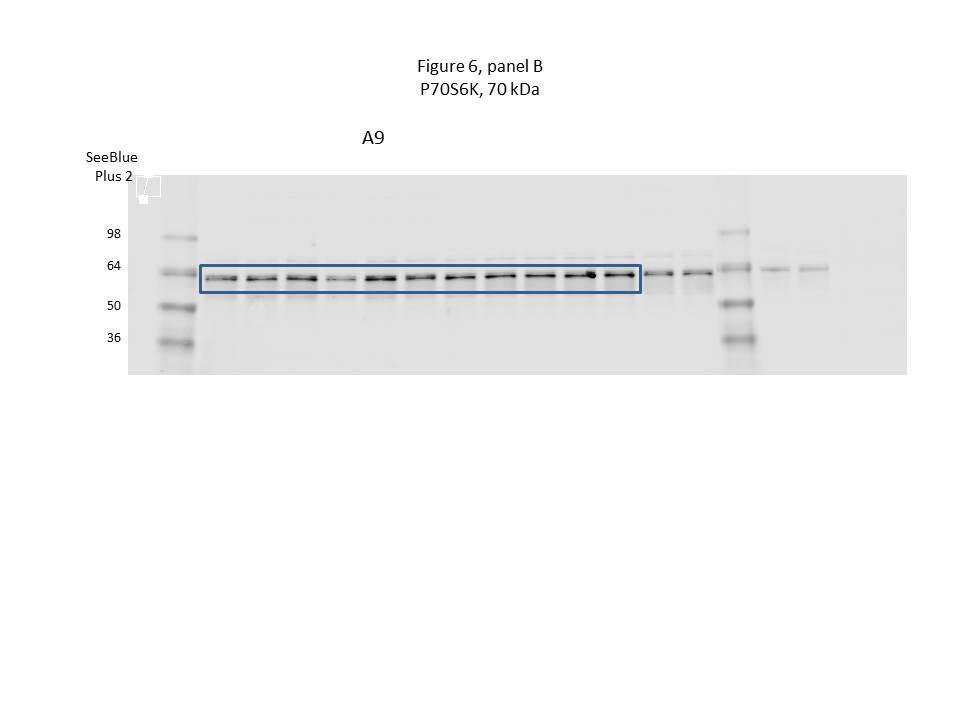

Supplement: Supplementary file 1 — Supplemental information [file 41598_2018_33453_MOESM1_ESM.doc]
